# Crimean–congo haemorrhagic fever virus circulates within broad ecological networks of ticks and vertebrates

**DOI:** 10.1371/journal.pntd.0013783

**Published:** 2026-05-27

**Authors:** Agustín Estrada-Peña, Oliver Carnell, Sara R. Wijburg, Maya Holding, Hein Sprong

**Affiliations:** 1 Department of Animal Health, University of Zaragoza, Zaragoza, Spain (retired); 2 Virology and Pathogenesis Group, Public Health Microbiology, UK Health Security Agency, Porton Down, United Kingdom; 3 Centre for Infectious Disease Control, National Institute for Public Health and the Environment (RIVM), Bilthoven, the Netherlands; 4 Laboratory of Entomology, Wageningen University and Research, Wageningen, The Netherlands; Medizinische Universitat Wien, AUSTRIA

## Abstract

We produced spatial datasets of the known distribution of *Orthonairovirus haemorrhagiae* (formerly Crimean-Congo haemorrhagic fever virus, CCHFV) by compiling human cases, virus isolations, and serological data from humans and animals, spanning from Europe to southern Africa, with the aim of virus range modelling. Models based solely on climatic variables produced unrealistic and overly extensive predictions, overestimating suitability in northern Europe and confirming that climate alone poorly explains CCHFV range. Approaches using only tick species distributions underperformed in many parts of Africa, reflecting the complexity of vector–host interactions and the absence of a strict tick–virus association. The integration of human-biting tick distributions, livestock density, and chorotypes yielded the most robust results, accurately capturing over 90% of known occurrences, by identifying vertebrate assemblages most likely to amplify the virus while reducing the number of explanatory variables. Chorotypes, representing clusters of co-occurring hosts, enhanced model performance and improved the delineation of both the Palearctic and African ranges limits of the virus. Our results support a generalist epidemiological model in which multiple tick species, together with a broad range of vertebrate hosts, sustain CCHFV circulation. This ecological flexibility, rather than strict vector specificity, likely explains the CCHFV wide biogeographical range and occasional lineage expansions across continents. This study provides the most comprehensive assessment to date of the determinants of the distribution of CCHFV. Although gaps remain, this study demonstrates that coupling abiotic and biotic predictors provides a more accurate and ecologically meaningful understanding of CCHFV distribution.

## Author summary

Crimean–Congo haemorrhagic fever virus (CCHFV) is a tick-borne pathogen causing severe disease in humans across Africa, Asia, and Europe. Understanding where the virus may circulate is crucial for anticipating outbreaks and guiding surveillance. Previous studies have mostly relied on climate-based models, assuming that temperature and humidity determine where infected ticks can prevail. However, such models often overestimate risk, predicting virus presence in regions where no cases or vectors exist.

In this study, we combined climatic data with ecological information on ticks, livestock, and wildlife to create a more realistic prediction of CCHFV distribution. By incorporating host–vector interactions and vertebrate communities, our models accurately captured most known occurrences and revealed the ecological patterns underlying virus transmission. Our results suggest that CCHFV persists within broad networks of tick and vertebrate species rather than a single vector. This integrated approach offers a powerful framework to identify emerging risk areas and forms a good starting point to further improve our understanding of the ecological drivers of tick-borne viral diseases.

## Introduction

Crimean-Congo hemorrhagic fever (CCHF) is a human disease caused by infection with *Orthonairovirus haemorrhagiae*, a segmented RNA virus commonly referred to as Crimean-Congo haemorrhagic fever virus, CCHFV, [1]. The virus circulates silently in tick-vertebrate-tick enzootic cycles with a brief viremia in vertebrates and prolonged viral maintenance in ticks by transstadial and transovarial transmission, and, less efficiently, by venereal transmission. The virus is widespread in the Old World, with a patchy distribution, and so is the reported disease occurrence, with the highest incidence in parts of Africa and West and Central Asia.

The main route of infection to humans is through the bite or handling of an infected tick of the family Ixodidae [2], in addition to infection through contact with infected livestock blood and tissues. Vertebrates that enable tick feeding, but do not support the multiplication of the virus, are known as maintenance hosts, as they contribute to the maintenance of the tick population.

Vertebrates in which the virus proliferates and infects ticks during feeding are known as amplification hosts, as they amplify the number of infected ticks in the environment. Cattle, goats, sheep, and other large ruminants are amplification hosts and may infect ticks in the relatively short viremic period [3,4]. Small mammals, like rodents and hares on which immature ticks feed, have a short and transient viremia and are not considered very effective for a tick infection but support the feeding of tick immatures. Co-feeding transmission of CCHFV has been demonstrated in the laboratory [5], but this way of amplification has been ignored in further field studies. Birds are most likely not viraemic, but some migratory birds may carry infected ticks in their transcontinental trips from Africa to Europe or Asia [6]. Studies support the limited role of long-distance migratory birds harbouring CCHFV-infected ticks for the establishment of the virus in a new territory [7]. Other routes of transmission involve the human-to-human (infected people admitted into hospitals with low protection for nursing personnel), from livestock carcasses via aerosol transmission during slaughter, or from crushing ticks manually.

Laboratory tests are the most straightforward method to assess the ability of ticks to acquire and transmit the virus. A review [8] found significant variability in results when using different virus strains, vertebrate hosts, or tick species. Despite these limitations and the limited number of tick species studied, these findings suggest that ticks of the genera *Hyalomma*, *Amblyomma*, or *Rhipicephalus* are vectors and reservoirs of the virus, capable of transmitting it transstadially and sometimes also transovarially.

Laboratory vector competence studies enable the identification of ticks which can transmit the virus to hosts but cannot differentiate between primary and secondary vectors. Primary vectors of CCHFV are tick species which are necessary for maintenance of the circulation of the virus under natural conditions and transmit to humans. Secondary vectors of CCHFV are tick species which can acquire and transmit the virus but cannot maintain virus circulations under natural conditions in the absence of a primary vector.

Since the last comprehensive review on CCHFV [6], our understanding of the virus’s epidemiology has seen minimal progress. CCHFV has been detected in 39 different tick species, collected from various hosts, while questing, or under experimental conditions, including *Amblyomma*, *Dermacentor*, *Hyalomma*, *Haemaphysalis*, *Rhipicephalus*, and even several species of soft ticks [9]. Interpreting these findings remains complex because ticks may acquire the virus from the blood of vertebrate hosts but may not be competent in transmission to other vertebrates or humans. For example, the role of soft ticks in the transmission has been demonstrated in the laboratory [10] and CCHFV RNA detected in the ticks; however, this is not equivalent to transmission capacity [11]. Misconceptions about tick vectorial competence persist in studies aimed at evaluating the prevalence of the virus in ticks, humans, or animals [12].

Severe knowledge gaps remain in the ecology of CCHFV, regarding (a) which tick species maintain the circulation of CCHFV among amplification hosts, (b) which tick species infect humans, and (c) which vertebrates allow sufficient replication of CCHFV and feed ticks that could then be infected. To further complicate the matter, CCHFV can be genetically separated into six major clades or lineages: three predominantly found in Africa (lineages I–III), two in Europe (lineages V and VI), and one in Asia (lineage IV) [13]. Whether these lineages vary in their ability to infect different vertebrate or tick species (host range) or whether there are variations in the ability of ticks to transmit these different lineages is poorly understood but may have a major impact on the current distribution of CCHFV and its ability to spread.

The production of maps pinpointing the range of CCHFV has been addressed on several occasions [14–18]. While it seems unlikely, it is not yet proven whether the range of CCHFV is exclusively linked to the known distribution of certain tick species or to a pattern of climate features without other explanatory forces. It is also unclear if the presence and/or abundance of specific wildlife species could represent the known range of CCHFV. A list of animals recorded to be positive in serological tests has been compiled [19], which was significantly updated [9]. Additionally, it is suggested that peculiarities in ecological relationships between ticks and vertebrates may influence the spatial complexity of the virus distribution [20]. A potential approach to understanding the CCHFV circulation and identifying areas prone to exposure could involve simultaneous mapping of vertebrates and ticks, along with climate and other relevant information.

We anticipate that utilising all available sources of information could produce suitable maps of the CCHFV range. Mapping the relationships between climate, ticks, and vertebrates across large regions as zoogeographical realms could facilitate the acquisition of epidemiological information through statistical interactions. This approach would not only benefit human health by predicting areas of risk for humans but also contribute to a deeper understanding of the actors involved in virus circulation. Such a framework should be grounded in extensive datasets of tick and vertebrate ranges, as well as sensitive methods that capture ecological associations and transform occurrence overlaps into an epidemiological message.

This study aims to model the distribution of CCHFV in the Western Palearctic and Tropics. It uses large georeferenced datasets of molecular and serological data from animals and humans, known georeferenced clinical records of the virus, and the joint distribution of 82 tick species and 121 genera of vertebrates reported as either maintenance or amplification hosts. The study focuses on detecting and defining the hypothetical environmental niche for the virus. If this environmental niche exists, CCHFV could only occur within the niche’s spatial projection, regardless of the presence of ticks and vertebrates in the area. The study also aims to identify the epidemiological associations of the virus with primary and secondary vectors. Additionally, it explicitly tests whether CCHFV occurs in conjunction with biogeographical entities known as chorotypes that describe ranges of cooccurring ticks and vertebrates as biogeographical entities known as chorotypes. The data presented in this study are intended to support a comprehensive approach to simultaneously test the interactions of communities of ticks and hosts, climate, and livestock distribution to explain the geographic distribution of CCHFV.

## Methods

### Modelling framework

**Fig 1:**
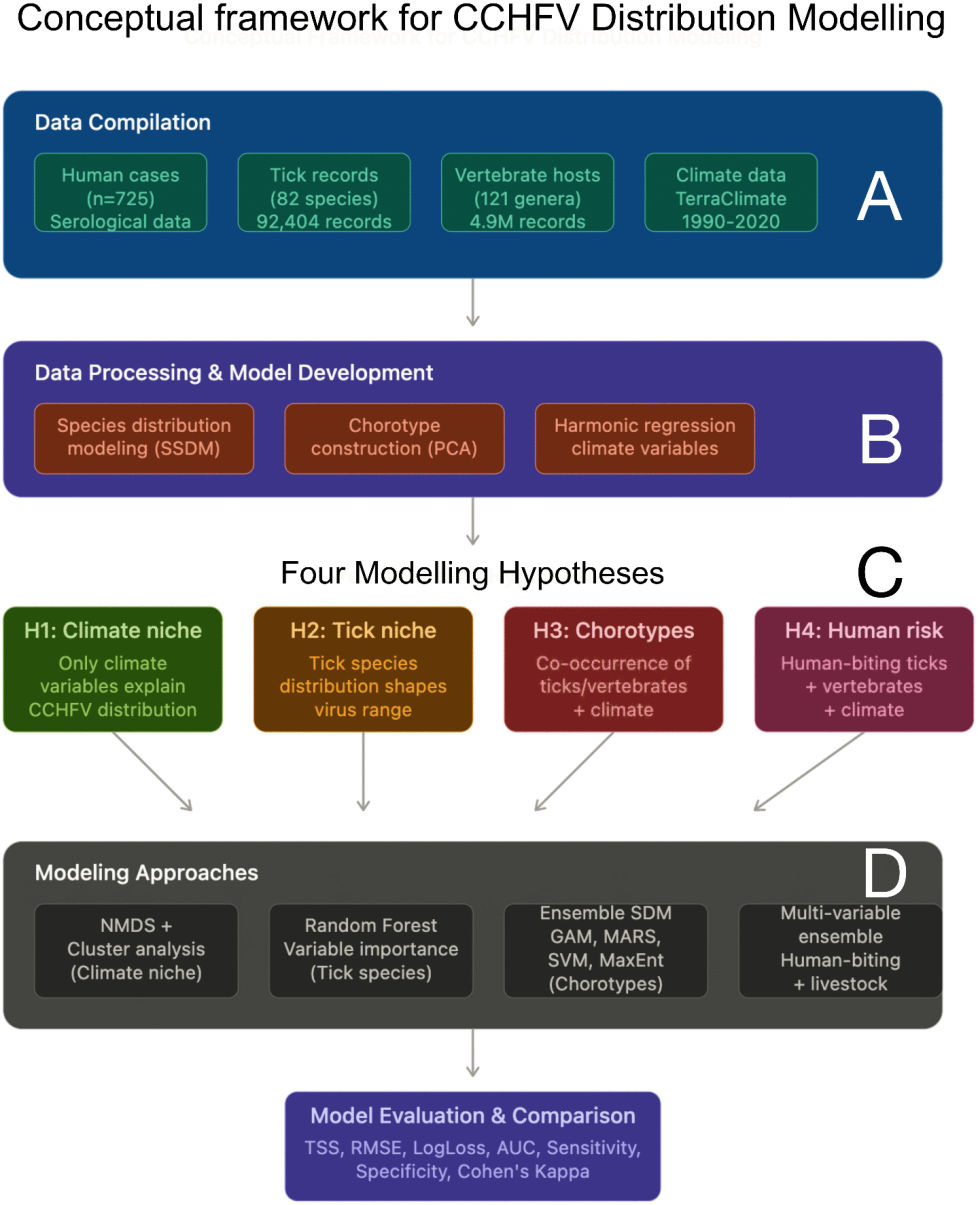
The conceptual framework of modelling framework for the distribution of CCHFV, aimed to show the steps in compiling data (A), data processing and model pre-development (B), the modelling hypothesis (C) and the final approach to the best model (D).

We explicitly modelled the known distribution of CCHFV, recorded as both human cases and serological surveys in humans and animals in a large territory. These spatial data are kept as the golden standard against which to contrast the reliability of the models. Fig 1 includes the schematic flow of hypotheses, data collection, and modelling steps. We used different methods adequate for each modelling strategy, that are detailed below.

We addressed four combinations of explanatory variables that could define the known range of CCHFV. Each one defines an ecological strategy of circulation of the virus and the specificity of the virus, being climate included in everyone as the main modulator of the distribution of the vectors and vertebrates that feed the ticks. These hypotheses are:

The distribution of CCHFV can be modelled only with climate features. This hypothesis supports that the only features that restrict the distribution of the virus are environmental.

The distribution of CCHFV can be modelled using the distribution of ticks that occur in the target territory. This hypothesis considers that the distribution of ticks is shaped by climate and that such as distribution delineates the range of the virus.

The distribution of CCHFV is better mapped using the climate features and the epidemiological constructs that result from the co-occurrence of ticks and vertebrates (as vectors of the virus and hosts for the ticks). This hypothesis supports the notion that only some combinations of vectors and vertebrates (plus the climate) can circulate the virus in foci.

Since humans are the only species that develops a clinical syndrome after infection with CCHFV, being serology a secondary detection method, it makes sense that the data on climate, distribution of human-biting ticks, and the associated mammals, may provide the best modelling approach.

### Area of study

**Fig 2:**
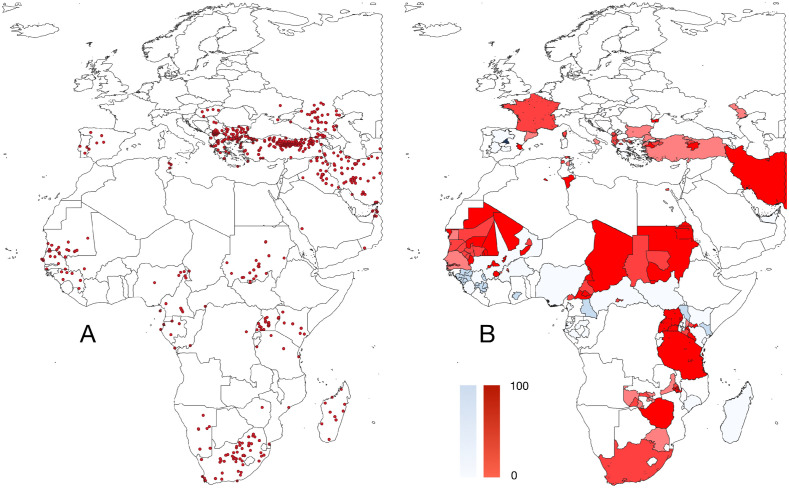
A. The 725 occurrences of Crimean-Congo haemorrhagic virus (CCHFV) detected in humans. B. The geographical distribution of the serological studies compiled from the literature, carried out in animals (tones of blue, 144 papers) or in humans (tones of red, 107 papers). The base layer of the maps was downloaded from https://www.naturalearthdata.com/downloads/50m-cultural-vectors/ and modified in qGIS.

The target territory includes the Western Palearctic and the Tropics, between the coordinates 72ºN, 36ºS, 58ºE, 18ºW. This includes all African and European countries, and western parts of Central Asia, approximately to the east limit of Iran (see Fig 2). The territories east of the Palearctic domain have not been included because of the lack of sufficient geo-referenced data. Findings of CCHFV in the area are commonly referenced to large administrative divisions (e.g. provinces, without coordinates), therefore preventing modelling. This study involves the range of both ticks and vertebrates as explanatory variables for modelling CCHFV extent, that needs coordinates. The inclusion of data (ticks records, human cases, etc.) without basic geo-referencing and at a medium resolution (e.g. the smallest administrative division) cannot be merged with the high-resolution framework developed for the rest of the target territory.

### Source of records and climate data

We consulted multiple sources to compile a reliable dataset of tick and vertebrate occurrences within the target territory. Tick records were sourced from three previous compilations [21,22], available data for specific tick species in Europe [23–25], and GBIF [26]. The list of ticks included in this study is available as Supplementary Material 1. For vertebrate distributions, we utilised two previously compiled datasets [21,22] to identify vertebrate species reported as tick hosts over the past 40 years. The list of vertebrates serving as tick hosts in the target territory was updated with published data [27] and additional data downloaded from GBIF [26].

Initially, the dataset included 132 tick species and 403 host species. We previously did modelling of every tick species (see below for details), checking against the recorded distribution of the virus. A spatial correlation was carried out between the layer of actual virus distribution and the model range of each tick species. Those with less than R^2^ < 0.2 were removed from further protocols. Records of *Haemaphysalis* spp. were removed after these initial tests because of the lack of correlation of these species with records of CCHFV, parasitism to humans, or solid links of ticks in the genus and transmission of the virus [8,9,19]. To address the logistic challenges posed by modelling over 400 species of vertebrates, we aggregated the original dataset to genera of vertebrates instead of species. The dataset of ticks was examined to eliminate species with less than 50 records. In total, we analysed a dataset containing 92,404 geo-referenced records of 82 tick species and 4,920,908 geo-referenced records of vertebrates across 121 genera.

### Climate data

Climate data were used to model environmental suitability for several groups of organisms across the target territory at a spatial resolution of 4 km/pixel. We sourced monthly data on maximum and minimum temperature and water vapour deficit from the TerraClimate repository (https://www.climatologylab.org/terraclimate.html, [28]) for the period between 1990 and 2020. For each month, we calculated the average values across the target territory using monthly values (1990–2020) and the “terra” package (v 1.8.70, [29]) for the R programming environment.

We performed harmonic regression on the 12 averaged monthly values (temperature or water vapour deficit) against time (in months, from 1 to 12). The coefficients derived from the harmonic regression were used as the explanatory variables for tick or vertebrate occurrence modelling [30]. These variables are orthogonal and thus non-self-correlated, eliminating the need to assess collinearity among variables for good modelling practices that irremediably removes variables of importance in tick ecology [31]. They also capture critical conditions for organism survival better than sets of pre-tailored climate data [31] because the complete series of daily data can be reconstructed from only the first three coefficients in the regression of each variable. Nine variables were used to describe climate, namely the first three coefficients of the harmonic regression for maximum temperature (TMAX), minimum temperature (TMIN), and vapour pressure deficit (VPD).

#### Modelling the distribution of ticks and vertebrates.

Since one of the aims of the study is to use ecologically sounding variables that can explain the range of CCHFV, we generated maps of the expected distributions of ticks and vertebrates. This is the obtention of new explanatory variables by modelling the known range of ticks and vertebrates, that were modelled using climate data. We used the approach known as stacked species distribution modelling (SSDM [32]). This method addresses the simultaneous evaluation of multiple algorithms on the complete set of species (either ticks or vertebrates), selecting the best combination through ensemble modelling (i.e., combining the results of various algorithms). The range of each organism was modelled using the known records, thinned to the 4 km resolution of the explanatory variables. We employed the following algorithms: generalised additive model (GAM), multivariate adaptive regression splines (MARS), and support vector machines (SVM) in the SSDM package (v. 0.2.11 [32,33]) in R. We also used the implementation of the algorithm MaxEnt in the package “Wallace” (v. 2.1.3 [34]).

For GAM, we ran 500 iterations per species/genus with an epsilon value of 1E-8; for MARS, we allowed up to 4 degrees of interactions; for SVM, we used the same epsilon as for GAM, with three cross-validation steps. For MaxEnt we optimised the parameters of feature classes and the regularization parameters, as recommended [34,35]. To select the best model parameters the feature classes were restricted to linear, quadratic, and product. The regularization multiplier values were 1, 2, and 5 [36]. Parameters not specified were kept at their default values in the package(s). In all cases, 70% of the records were used for model training and 30% for model testing (ten replicates each). Pseudo-absences were generated automatically by the software in a number proportional (x10) to the actual records of the organism to be modelled.

The metrics for selecting the best models for inclusion in the ensemble SDM were the threshold value (determined using the value that maximises the sum of sensitivity and specificity, also known as MaxSSS), a widely used criterion in species distribution modelling because it balances omission and commission errors and is independent of species prevalence), the omission rate (proportion of missing records with the best model), sensitivity (the proportion of correctly predicted presences), specificity (the proportion of correctly predicted absences), proportion of correctly detected records (overall accuracy). We selected to combine all the models that outcome a threshold above 0.7. The area under the curve (AUC) was used for model comparison, although its interest as a metric for model performance was restricted to comparisons within the same species. The complete set of maps for both ticks and hosts is available in FigShare at https://figshare.com/s/9ffe21744531bddf67db.

#### Building of chorotypes.

A chorotype can be defined as a distribution pattern that can be shared by a group of species or by a single species (monospecific chorotype). Chorotypes are the roots of an epidemiological regionalisation because they consist of the identification of spatial units with similar species composition [37]; in classic terminology, chorotypes can be considered clusters of co-occurring species, and as such detected. Their applicability for exploring epidemiological properties of co-occurring ticks and hosts in large territories was demonstrated before [38]. We applied this concept, separately, to the modelled distribution of either ticks or vertebrates, aiming to obtain a set of a few variables that can be used to predict the distribution of the virus. The rationale is that if ticks or vertebrates co-occur, the map(s) of distribution of these chorotypes summarise the information of dozens or hundreds of potential predictors, improving and shortening the modelling.

We applied a clustering procedure [39] based on Principal Components Analysis (PCA). Chorotypes resulted from optimising the number of clusters required to explain observed variability through the ‘elbow’ method in the package “recluster” (v 2.9, [40]). The aim of PCA was both to reduce the spatial variability into axes that explained variance and to define epidemiological spatial units (chorotypes) that will be used later as part of the set of explanatory variables used to capture the range of CCHFV.

#### Compilation of human cases.

Data on the geographical distribution of CCHFV were used to train the models predicting the distribution (probability of presence) of CCHFV. The reported cases of CCHFV in the target territory were obtained from compilations [15,16] that were complemented with known cases from Portugal, Spain, and Türkiye, not included in the mentioned compilations but reported previously [41] and with a few records available in GenBank with details to a locality of isolation. Most records of CCHFV in GenBank refer to large administrative divisions, commonly countries, and therefore were not included in this compilation, because the data reported to whole countries cannot be unequivocally ascribed to a pair of coordinates. The total number of georeferenced human cases and virus isolation was 725.

#### Compilation of serological data.

We compiled published data on serological surveys conducted in humans and animals by performing a comprehensive review of electronic bibliographic databases for English-language journals. The serological survey data served as an additional source for training the models of CCHFV distribution and are available in Supplementary Material 2.

We used Pubmed, Scopus and web of science from inception to September 2024 inclusive using the following search terms: CCHF, CCHFV, Crimean hemorrhagic fever, Crimean haemorrhagic fever, Congo hemorrhagic fever, Congo haemorrhagic fever, Crimean-Congo haemorrhagic fever, serological, serosurvey, serosurveillance, sero surveillance, seroprevelance, seropositivity, seroepidemiologic, seroepidemiology. This search gave 423 published studies. The results were further restricted to those relevant to Africa, parts of Asia, and Europe, to overlap the target territory. Studies were rejected in instances where the full text was unavailable, not available in English, or where the focus was on other viruses or the papers were focused on the development of methodology. Only studies with appropriate spatial information were included (see below for spatial processing of information). This resulted in 76 papers with human data and 104 papers with animal serology data, for which we extracted the serological rates.

For papers containing suitable data, we recorded the following information: DOI, first author, title, year of publication, country, region of interest (e.g., a province, region, etc.), and the Global Administrative Areas (GADM) level 3 administrative division. GADM level 3 is equivalent to the municipalities (NUTS3) in Europe and is available at https://gadm.org.

We also included the year the study was conducted, the number of people (or animals) involved in the serological study, their occupation, whether tick bites were recorded, and whether they had contact with animals. Additionally, we compiled data on seroprevalence, specific IgG and IgM values, the test used for the serological survey, and the confirmatory test for the virus. If sequencing was performed, we included the GenBank accession number of the sequence(s). We included details of environmental factors, presumed risk factors, and how these factors were statistically evaluated. In the case of animals, we also included details about collections of feeding ticks and if the virus was detected in these ticks. Notably, most papers did not include all the attributes mentioned above. We did not remove papers missing one or several components to ensure a complete dataset of serological surveys conducted for CCHFV. Data on (infected) ticks feeding on animals were not included in the database as those infection rates give unreliable representation of viral prevalence in ticks.

The inclusion of serological data for either animals or humans posed a challenge in terms of homogenisation. The literature search revealed a diverse range of formats. Serological data was most reported as data for administrative divisions of varying sizes, always smaller than the country, but rarely was data presented with spatial coordinates or the name of a locality that could be accurately referenced to a pair of coordinates. To address this issue, we simplified the original dataset of serological data to a common resolution, focusing on the smallest administrative divisions. Subsequently, we employed a tessellation method, which has proven effective in similar problems [22]. This method involved covering the entire target territory with hexagons, each with a radius of 2º. Each hexagon was then overlaid with the serological rates for either animals or humans using GIS software’s overlap routines. The values of serology, after being reported and transformed to the smallest administrative division, were assigned to the corresponding hexagon. The centroid of each hexagon within the honeycomb, where positive serological rates were detected (either for humans or animals) or where molecular tools indicated the presence of the virus, was considered the geo-referenced point for further modelling.

For most modelling protocols of the distribution of CCHFV, we used a set of pseudo-absences. This set replaces the true absences of the virus, that are commonly not reported, and complements the data of 0% seroprevalence in either animals or humans. The set of pseudo-absences was generated using a script in R that randomly generates pairs of coordinates, selects those that are on the ground (to remove those that are located over the sea) and that are separated from true records of the virus by at least 4 km, because this is the resolution of all the raster layers. The set of random pseudo-absences and the true serological absence of CCHFV are 1,217 points. The range of serological surveys in either humans or animals has been included in Fig 2.

This protocol was impossible to apply to existing data from countries approximately east to the right margin of the target territory. Data on tick records and/or CCHFV exist, but (a) ticks are not recorded by coordinates (but administrative units, particularly in China and Russia), results have been published in national journals (in Chinese or Russian), and serological data for humans and/or animals follow the same reporting scheme along administrative regions. So, it is known that CCHFV exists there, but the existing data are unsuitable for modelling.

### Modelling the range of CCHFV

After the compilation of climate data, the records of ticks and vertebrates, their modelled ranges and the chorotypes resulting from species co-occurrence, we modelled the distribution of CCHFV, using several strategies that we explain below.

#### The climate niche of CCHFV: modelling only with climate variables.

We first determined if CCHFV has a climate niche. This was done by analyzing the 725 human case records and the 1,217 pseudo-absences records placed over the target territory, against the nine environmental variables obtained as coefficients of the harmonic regression of TMAX, TMIN, and VPD. Climate information for each presence/pseudo-absence point was extracted. Next, we subjected the set of points to a Non Metric Multidimensional Scaling (NMDS). NMDS is a gradient analysis approach that uses ordination [42]. It compares the pairwise dissimilarity between the climate covariates of viral records using their variation among sites. Each record of CCHFV is assigned a pair of “coordinates” resulting from the NMDS, which provides it with a “position” in the classification space. The set of data is then subjected to a cluster analysis using kmeans. If the cluster analysis can statistically (a) separate negative and positive records, and (b) group all the presence points into a well-defined cluster, it is indicative of a climate niche for CCHFV because presences and pseudo-absences can be separated with an assumable error by the values of the environmental variables. Conversely, if the cluster analysis cannot resolve a single group of records, it is suggestive of the existence of multiple climate regions where CCHFV can thrive; in the later, it would be not recommendable to map the probability of distribution of CCGFV using only climate variables.

#### The “tick niche” of CCHFV: modelling with the range of individual species of ticks.

We modelled the distribution of CCHFV using only the tick distribution, without any prior assumptions about their vectorial capabilities. This approach suggests that any tick species could be involved in CCHFV circulation, and ranges of several species may overlap within the known CCHFV We included this part of modelling because it is the classic approach followed in previous studies, i.e. the distribution of the virus is shaped exclusively by the distribution of ticks. range.

It is impractical to model the extent of CCHFV using raster layers representing the hypothetical involvement of 82 species of ticks, entered one by one, in pairs, triads, and so on (this represents the factorial of 82 species in terms of possible combinations). We employed a Random Forest (RF) framework to evaluate the relative contribution of each tick to the potential distribution of the virus. RF is an ensemble learning method based on the construction of many decision trees, each trained on a bootstrap sample of the original dataset [43]. Predictions from individual trees are aggregated to produce robust and stable estimates, reducing overfitting and capturing non-linear relationships between predictors (the tick ranges) and the response variable (presence/absence of CCHFV).

We trained the RF using the observed presence and pseudo-absence of viral cases as the binary response (725 points of CCHFV presence, and the set of 1,217 pseudo-absences) and tick species ranges (raster layers) as predictor variables. Each tree in the forest was grown without pruning, and a subset of predictors was randomly sampled at each split to decorrelate trees and improve generalisation. Variable importance was assessed using permutation-based measures, which quantify the decrease in predictive performance when a given predictor is randomly permuted. The RF model outputs include the predicted probabilities of CCHFV occurrence. We assumed multiple tick species might be positively correlated with the known CCHFV range, so we produced a rank of each tick in the model CCHFV-ticks.

We calculated the True Skill Statistic (TSS), which balances both omission (false negatives) and commission (false positives) errors. A high TSS indicates that the model accurately identifies both presences and absences. It ranges between -1 and 1, where 1 is perfect agreement between predictions and observations, and <0 is worse than random. The Root Mean Squared Error (RMSE) measures the average magnitude of prediction errors. Lower RMSE means the predicted probabilities are closer to the observed outcomes. We calculated the LogLoss (Logarithmic Loss / Cross-Entropy Loss), which penalises confident but incorrect predictions more heavily than small errors. It is particularly sensitive to probabilities near 0 or 1 that are wrong. In the context of ecological modelling, a low LogLoss indicates that the model not only classifies correctly but also assigns accurate probabilities of presence, which is critical when making spatial risk maps. Values near zero indicate perfect predictions.

#### The “chorotype niche” of CCHFV: with chorotypes of ticks or vertebrates.

We assumed that the previous modelling protocols may be simplistic representations of the range of CCHFV and aimed to explore various combinations of other explanatory variables. In each instance described below, we employed the algorithms GAM, MARS, SVM, and MaxEnt, with the same parameter tuning as before. The metrics used to select the best models for inclusion in the ensemble SDM included the threshold value (the value that maximises the sum of sensitivity and specificity), the omission rate (proportion of missing records with the best model), sensitivity (proportion of correctly predicted presences), specificity (proportion of correctly predicted absences), overall accuracy (proportion of correctly detected records), and Cohen’s Kappa. In the case of the chorotypes as descriptive variables, we assumed that they encapsulated the redundant information contained in the raster ranges of several species, thereby decreasing the number of variables entered. Here, our hypothesis was that if a chorotype is positively associated with the occurrence of CCHFV, then some ticks/hosts in the chorotype may be involved in the transmission or amplification of CCHFV.

The combinations of variables were:

1) **The chorotypes of ticks or vertebrates, included *separately* as independent sets of variables, plus climate variables**. This approach was intended to capture the joint effect of co-occurring ticks or vertebrates as explanatory variables of the CCHFV occurrence. No single organisms were separately introduced in the model. The aim was to demonstrate that CCHFV occurs under critical combinations of hosts or ticks.
2) **The distribution of human-biting tick species, the distribution of chorotypes of vertebrates, and the density of livestock, plus climate variables.** This approach was intended to evaluate the distribution of human-biting ticks, the distribution of hosts (as their chorotypes), and the density of livestock as explanatory variables. The rationale was that most of the transmission events of CCHFV are derived from tick bites to humans; some cases are known to be produced by aerosol transmission of the virus during peridomiciliary slaughtering or bushmeat practices.

The ticks included in this analysis as human-biting ticks were *Amblyomma hebraeum, Amblyomma variegatum, Dermacentor marginatus, Dermacentor reticulatus, Hyalomma aegyptium, Hyalomma anatolicum, Hyalomma excavatum, Hyalomma marginatum, Hyalomma rufipes, Hyalomma scupense, Hyalomma truncatum, Hyalomma turanicum, Ixodes ricinus*. Among these ticks, some are known to transmit CCHFV in the laboratory [8], while others have been repeatedly reported as parasites of humans [44] but have not been explicitly tested as vectors of CCHFV. The *Rhipicephalus sanguineus* group, known to bite humans, was excluded from this analysis because the probable misidentification of many specimens in the large range could bias the information about species that bite humans against those that do not. The information about livestock density was obtained from the world map of livestock provided by the Food and Agriculture Organisation (FAO) (https://www.fao.org/livestock-systems/global-distributions/en/) and entered as three separate layers for cattle, sheep, and goats.

## Results

### 1). Models of ticks and vertebrates and chorotype outlining as explanatory variables of CCHFV

Before reporting the results on the different strategies of CCHFV modelling, we consider it necessary to show the results about tick, vertebrate, and chorotype modelling, because they will be used in the combinations of explanatory variables for CCHFV modelling. The results focus on the performance of models to obtain the explanatory variable that will be use later for CCHFV modelling.

The performance metrics of the algorithms used to model the distribution of ticks and vertebrates are included in Table 1. Values represent the averages across the full dataset of either ticks or vertebrates, and do not refer to a particular species. Overall, model performance was consistently higher for ticks than for vertebrates, despite the substantially larger number of vertebrate records relative to those of individual tick species.

**Table 1:** The performance indexes for each algorithm for the predictive mapping of either ticks or vertebrates, that will be used as explanatory variables of CCHFV occurrence.. These parameters include the threshold (optimised for sensitivity and specificity to produce a suitable habitat threshold), AUC (Area Under the Curve), the omission rate (proportion of presences classified as absences), sensitivity (model’s ability to detect positive instances), specificity (proportion of true negatives correctly identified), accuracy (percentage of correctly classified records, either presence or pseudoabsence), and Cohen’s Kappa (agreement between observations and predictions).

Algorithm	Threshold	AUC	Omission rate	Sensitivity	Specificity	Accuracy
Ticks						
MAXENT	0.2805	0.9578	0.4677	0.9012	0.5298	0.5322
GAM	0.5974	0.9639	0.0866	0.9136	0.9130	0.9133
MARS	0.6305	0.9322	0.0981	0.9034	0.9049	0.9082
SVM	0.7736	0.9476	0.1008	0.9001	0.8982	0.8991
MAXENT	0.4360	0.8623	0.2151	0.7853	0.7847	0.7849
GAM	0.3970	0.8758	0.2039	0.7954	0.7967	0.7961
MARS	0.4350	0.8492	0.2152	0.7853	0.7843	0.7848
SVM	0.4207	0.9467	0.4249	0.8652	0.5699	0.5750

For ticks, the modelling generated predictive maps for 82 species, with generally strong performance (mean sensitivity = 0.91; mean specificity = 0.91). Generalised Additive Models and MARS achieved the highest accuracy, whereas SVM performed slightly less effectively. Notably, the models developed using Maxent showed a poor balance between omission rate, specificity, and accuracy, as evidenced by their high omission rate. For vertebrates, the modelling of the known distributions yielded maps for 121 genera, with more modest results (mean sensitivity = 0.73; mean specificity = 0.73). Among the algorithms, MARS, GAM, and SVM performed better than MaxEnt, which outputted high omission rates. Notably, the lowest proportion of correctly allocated records of vertebrates occurred with SVM. The final output of the maps of each species/genus was prepared with the best combination of algorithms and variables, obtained, as explained above, by the best value software AUC, threshold, and accuracy.

**Fig 3:**
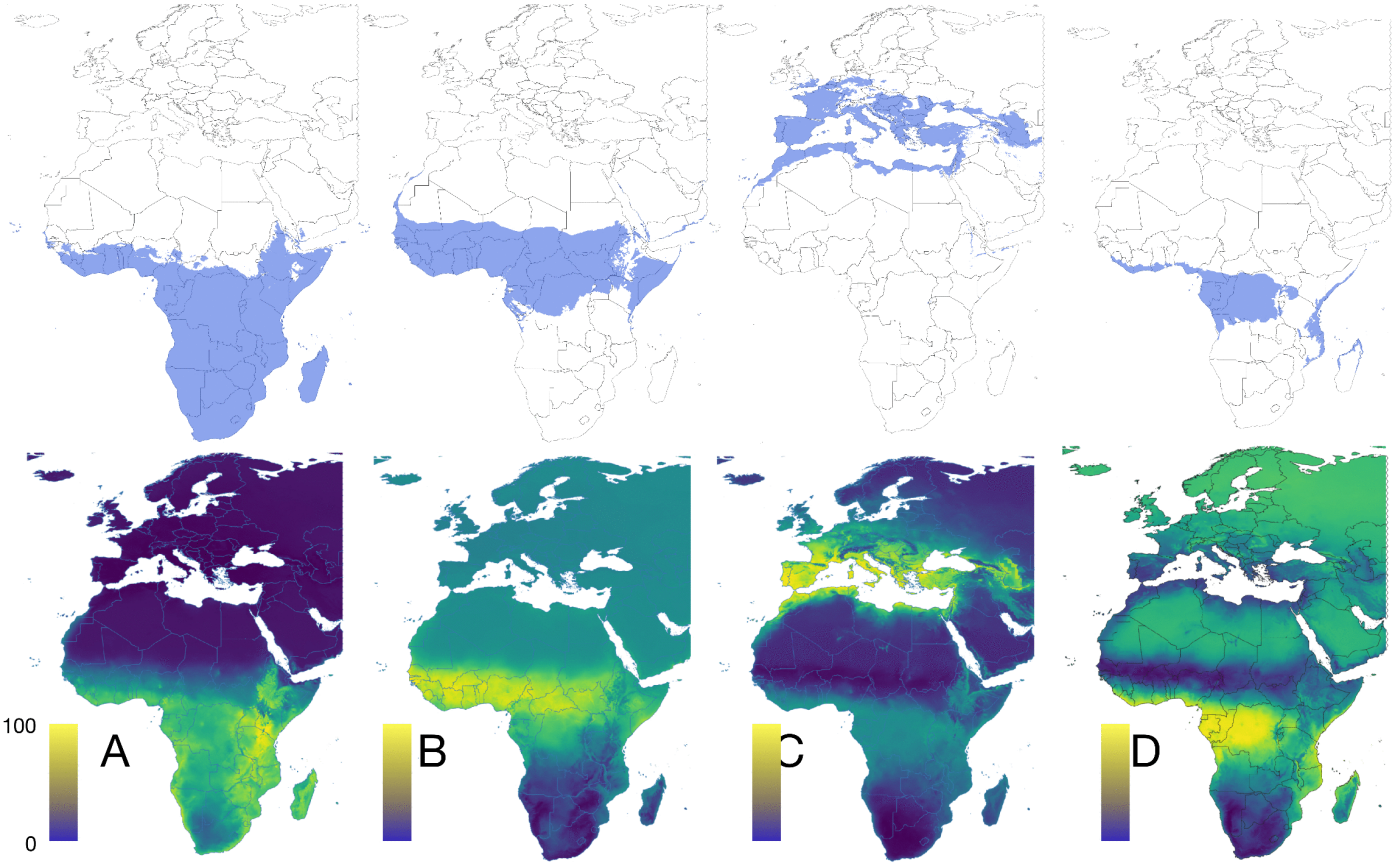
The chorotypes of tick species in the target territory, consecutively named A to D. Maps in the top row display the range of binomial positive suitability (blue). Maps in the bottom row display the probability of occurrence of each chorotype in a range normalised between 0-100. The threshold for binary occurrence has been calculated from the sensitivity and specificity of the predictive mapping of ticks in each chorotype. S2 Fig includes the loadings of each tick species in each chorotype. The base layer of the maps was downloaded from https://www.naturalearthdata.com/downloads/50m-cultural-vectors/ and modified (raster and additional vectors) in both R and qGIS.

**Fig 4:**
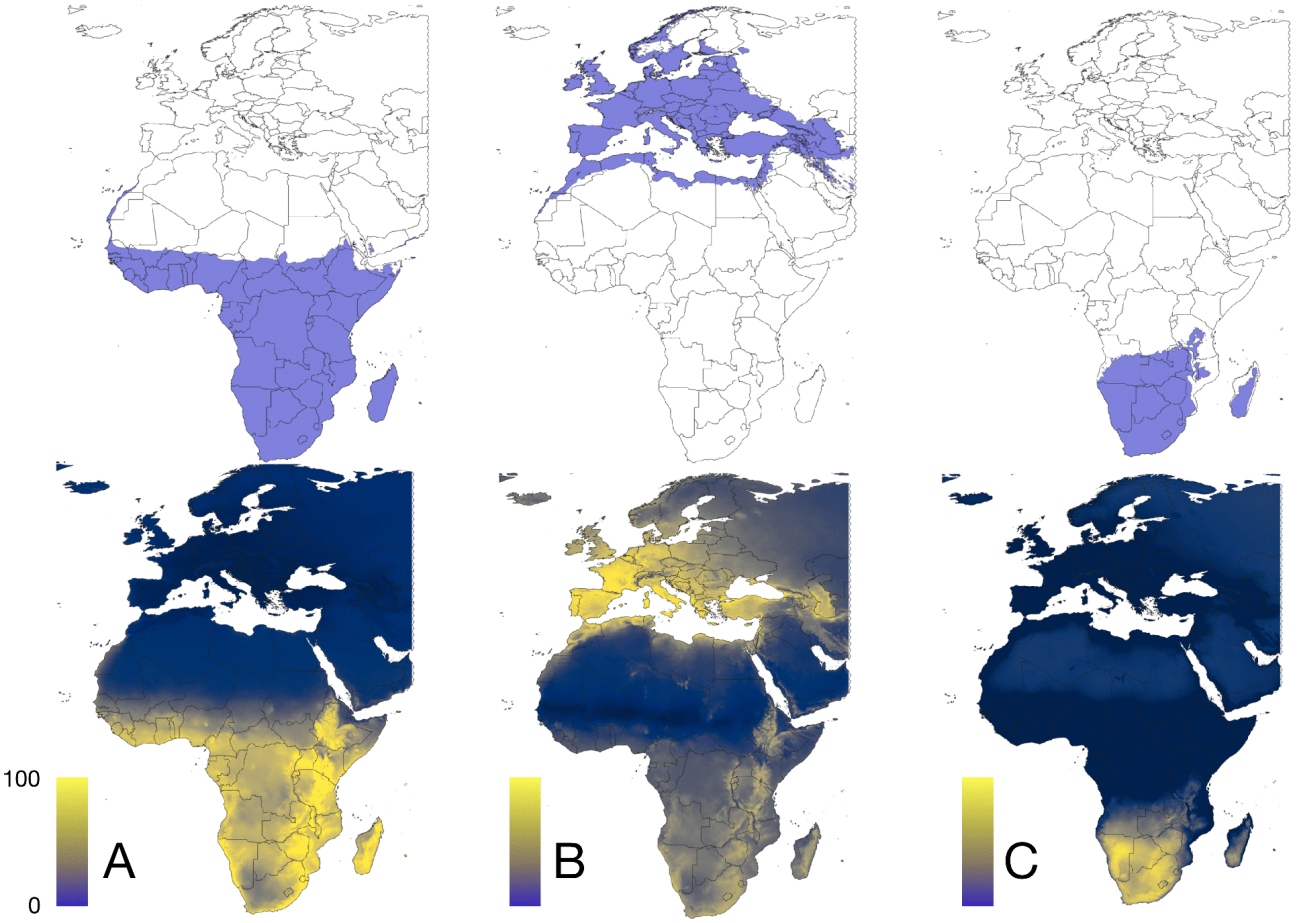
The chorotypes of vertebrate genera in the target territory, consecutively named A to C. Maps in the top row display the range of binomial positive suitability (blue). Maps in bottom row display the probability of occurrence of each chorotype in a range normalized between 0-100. The threshold for binary occurrence has been calculated from the sensitivity and specificity of the predictive mapping of ticks in each chorotype. S2 Fig includes the loadings of each vertebrate species in each chorotype. The base layer of the maps was downloaded from https://www.naturalearthdata.com/downloads/50m-cultural-vectors/ and modified (raster and additional vectors) in both R and qGIS.

Chorotypes are synthetic expressions of the co-occurrence patterns of either ticks or hosts, that explain the range of several taxa with similar distributions, detecting even groups of organisms that co-occur "inside" other chorotypes. The original 82 distribution maps of ticks were reduced to four significant chorotypes (Fig 3), jointly explaining 81.24% of the total variance (49.26%, 14.65%, 10.03%, and 7.37%, respectively). The ordination of the genera of vertebrates along the first PCA-derived axes is shown in S2 Fig. The original 121 maps of vertebrates were reduced to three chorotypes (Fig 4) that explained 74.22% of the variance (47.24%, 20.86%, and 6.12%, respectively). The ordination of the genera of vertebrates along the first PCA-derived axes is shown in S2 Fig.

### 2). Modelling the range of CCHFV

#### 2.1). A climate niche for CCHFV could not be detected.

**Fig 5:**
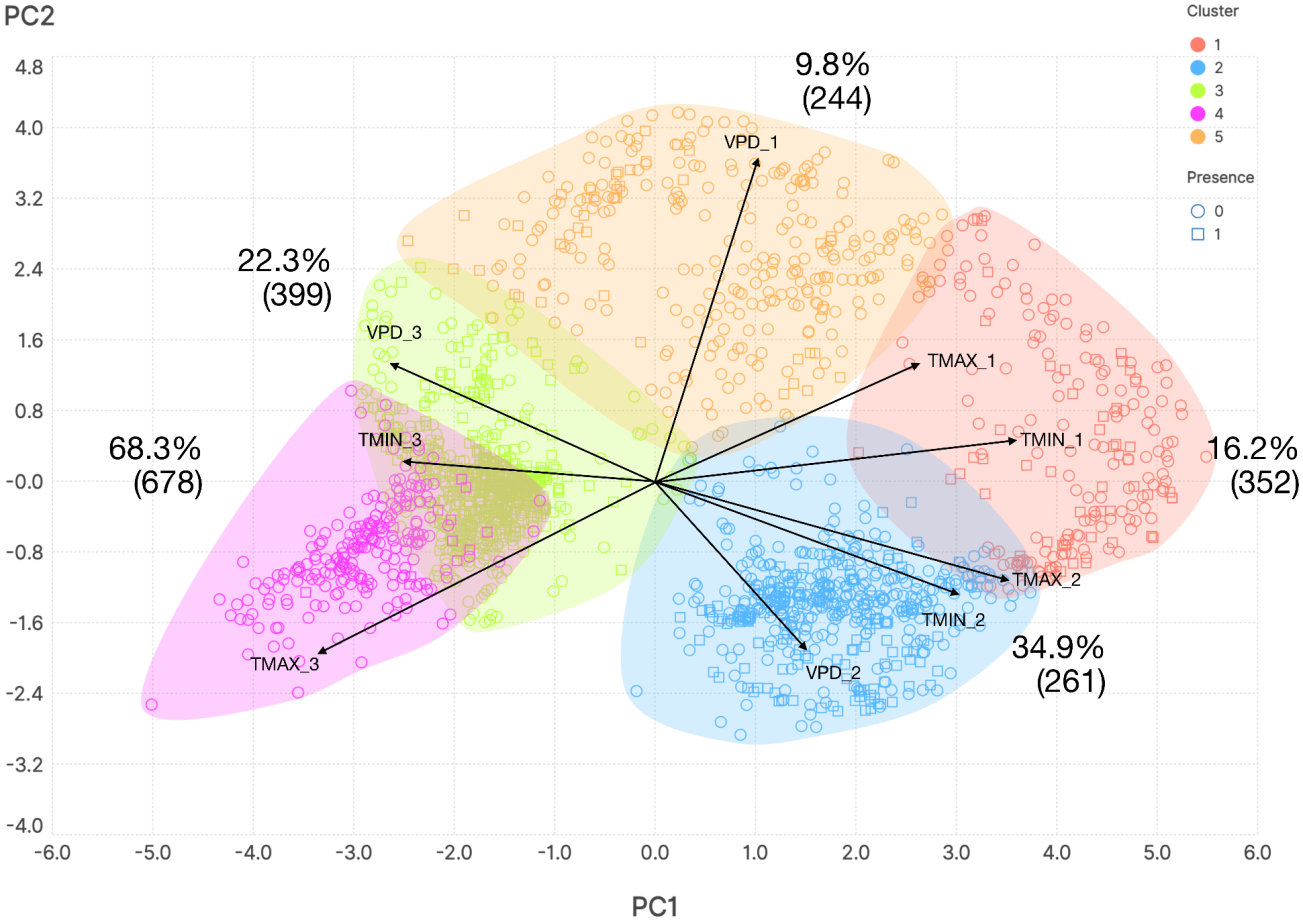
The plot of a non-metric multidimensional scaling (NMDS) in the reduced space, showing 725 records of CCHFV presence and 1,217 pseudo-absences. The NMDS was trained with climate variables to discern a hypothetical climate niche of CCHFV. The optimised output consists of five clusters that explain the climate variability. Positive and negative records of CCHFV could not be separated by the method.

The Non-metric Multidimensional Scaling (NMDS) analysis of 725 points of CCHFV presence and 1,217 pseudo-absences revealed five clusters of CCHFV records based only on climate covariates across the study region (Fig 5). NMDS failed to discriminate between presence and pseudo-absence points, as all clusters contained both categories in variable proportions. Cluster 4 contained the highest proportion of positive records (68.3%) in the entire dataset, but these were not correctly segregated from pseudo-absence points. The lack of clear separation between presence and pseudo-absence records strongly suggests the absence of a climate niche for CCHFV. Instead, the known range of CCHFV appears to exhibit a patchy distribution that cannot be adequately explained by climate variables alone.

**Fig 6:**
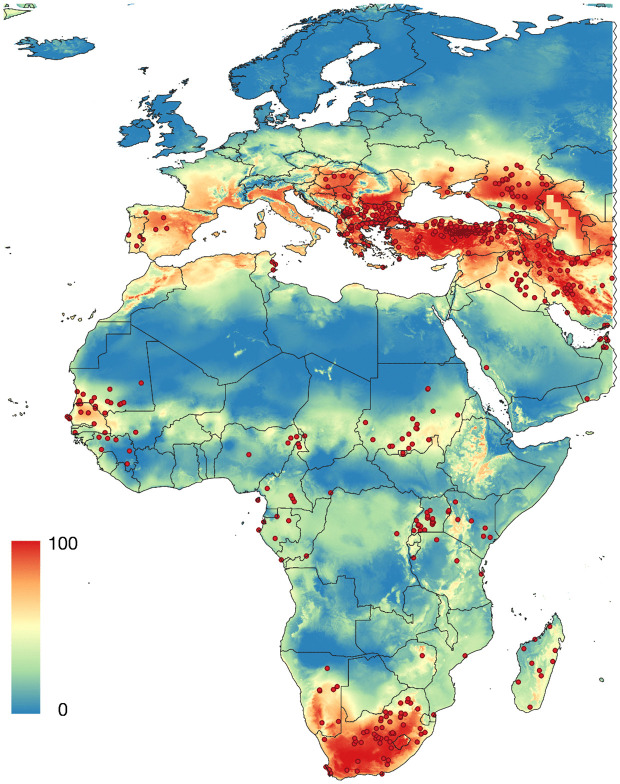
The probability of occurrence of CCHFV calculated only with climate variables (derived from maximum and minimum temperature, and vapour pressure deficit), using a stacking species distribution model (SSDM). The base layer of the maps was downloaded from https://www.naturalearthdata.com/downloads/50m-cultural-vectors/ and modified (raster and additional vectors) in both R and qGIS.

To further explore this, we projected the distribution of CCHFV using only climate variables with the set of algorithms explained in the methods. The best-performing ensemble from the SSDM modelling (Fig 6) predicted high probabilities of occurrence in the Mediterranean and South Africa. However, the threshold values were unacceptably high, ranging from 0.22 (SVM) to 0.45 (MARS) and 0.44 (GAM).

The combined model based on the best algorithm, tended to overpredict. This resulted in areas of apparent high suitability for CCHFV where no records of the virus or positive serology data exist. Consequently, extensive regions of false positives were found in central Europe, where no records of human cases are known to exist. The specificity values of the best model remained below 0.18, with high omission rates of 0.22–0.31. In other words, this approach failed to capture the known distribution of CCHFV in large parts of the Western Palearctic and the Tropics. Therefore, climate alone is not suitable for modelling the distribution of CCHFV.

#### 2.2.) Modelling CCHFV occurrence only with the tick ranges outcomes poor results.

We employed a Random Forest (RF) framework to evaluate the relative contributions of different ticks to the potential distribution of the virus. This is a practical solution to the very large computational task of modelling the range of an organism through the combination of occurrence layers of 82 species of ticks, that must be entered in many combinations, until the best model is obtained.

**Fig 7:**
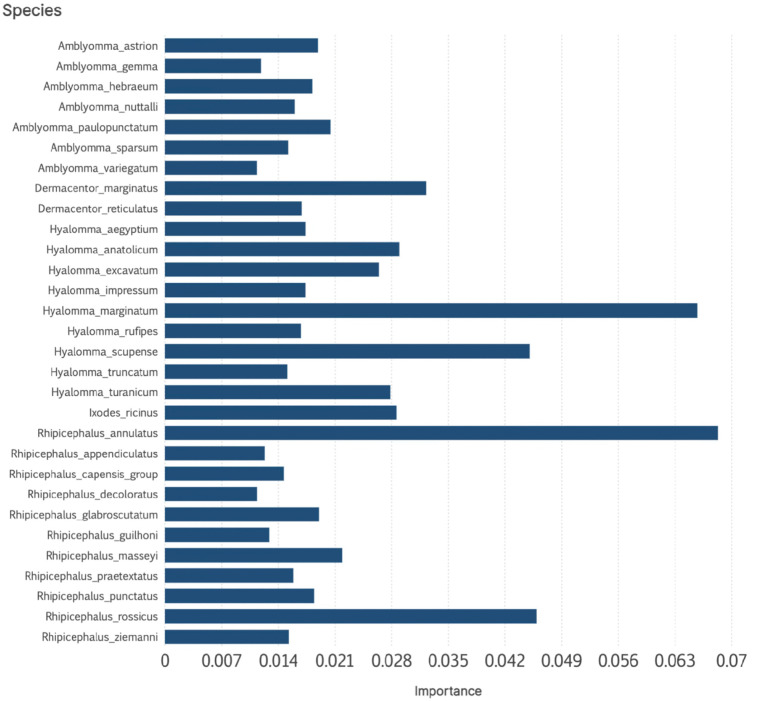
The relative importance of 20 species of ticks detected as the species that are positively correlated with the distribution of CCHFV, when used as explanatory variable for a model based on Random Forest. Notably, the list is composed by all the ticks that bite humans in the target territory, including all the *Hyalomma* spp. that bite humans, plus a selection of ticks that may be important parasites of Artiodactyla.

The RF model correlating available records of CCHFV against the range of the ticks produced good performance values, with a TSS = 1 (the 100% of 1,942 presence/absence of the virus were correctly allocated), RMSE = 0.1434 and LogLoss = 0.1063. Other values of performance of the best model were sensitivity = 0.82, specificity = 0.87, accuracy = 0.84, and Cohen’s kappa = 0.67. The model detected 20 species that are positively correlated with the distribution of CCHFV, included in Fig 7, together with its relative importance as explanatory variable for the model. Notably, the list is composed by all the ticks that bite humans in the target territory, including all the *Hyalomma* spp. that bite humans, plus a selection of ticks that may be important parasites of Artiodactyla. Other species, like *A. sparsum* or *H. aegyptium* parasitize also reptiles but are in the list of important explanatory variables. The importance of one-host ticks like *R. annulatus* in the model is also noticeable.

**Fig 8:**
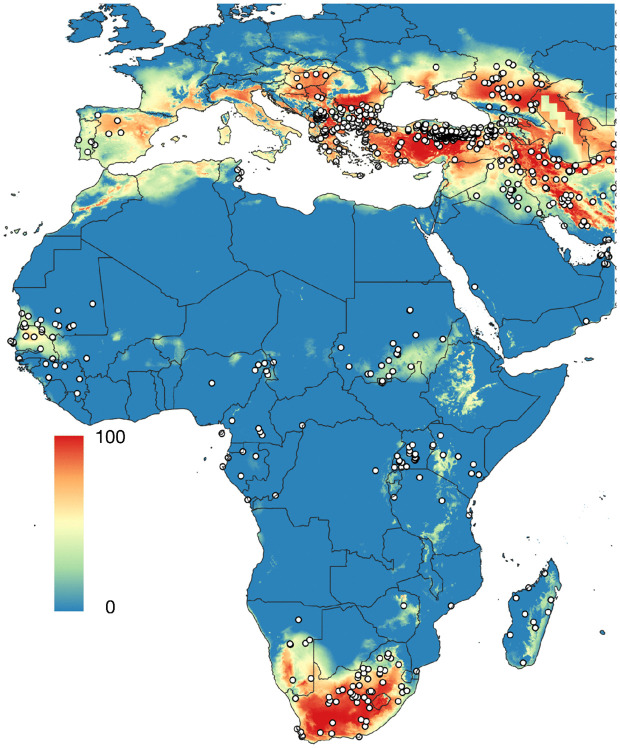
The probability of occurrence of CCHFV using the distribution of the ticks, used individually as explanatory variables. The base layer of the maps was downloaded from https://www.naturalearthdata.com/downloads/50m-cultural-vectors/ and modified (raster and additional vectors) in qGIS.

We further projected the RF model using the ranges of the ticks above as explanatory variables to draw the expected range of CCHFV (Fig 8). Being statistically solid as explained before, the RF model detects correctly the known distribution of the positive and negative records. However, the model is restricted to the positive sites of the training set, and the occurrence of CCHFV is projected only for sites where the virus has been already detected; there are not estimations of positive suitability out of positive site used for training. We consider that, being a robust algorithm, the choice of the tick ranges as explanatory variables is not suitable for acurately modelling the distribution of CCHFV.

### 3). *CCHFV occurrence is better explained combining tick* or *host chorotypes*

**Fig 9:**
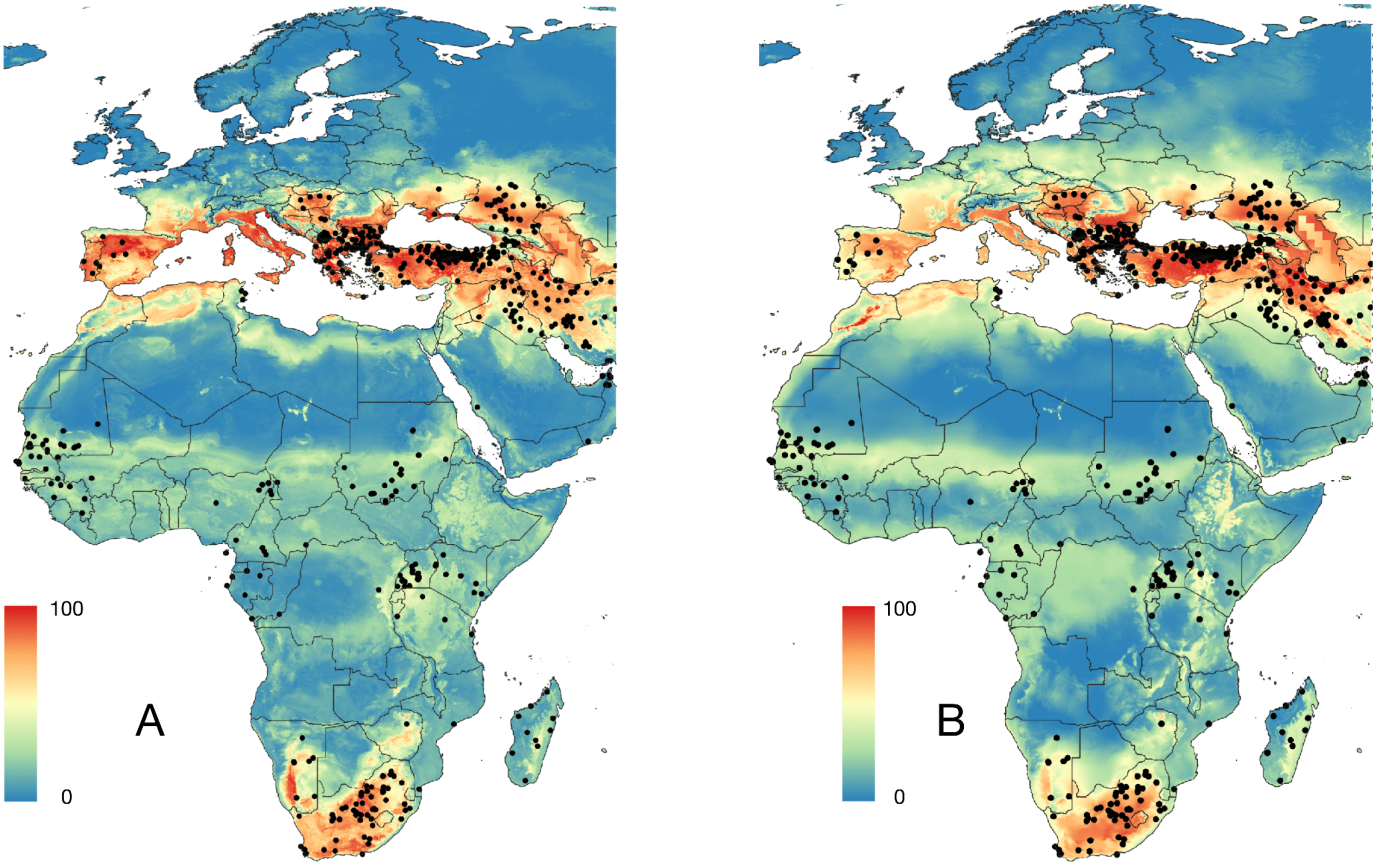
A. Probability of occurrence of CCHFV calculated with the chorotypes of vertebrates and the climate variables. B. The probability of occurrence of CCHFV using the chorotypes of ticks and the climate variables as explanatory variables, trained with the points of CCHFV presence and pseudo-absence. The base layer of the maps was downloaded from https://www.naturalearthdata.com/downloads/50m-cultural-vectors/ and modified (raster and additional vectors) in qGIS.

We tested more complex models integrating additional explanatory layers. Fig 9 presents three approaches: tick chorotypes (Fig 9A), or vertebrate chorotypes (Fig 9B).

Models using chorotypes alone (either tick or vertebrate-derived) performed worse, tending to overprediction in the case of the vertebrates as explanatory variables, suggesting that the information from chorotypes was insufficient to capture the full complexity of CCHFV ecology and may obscure critical epidemiological relationships. Using the chorotypes of vertebrates, the best model produced a map in which large portions of Central Europe, Ukraine, or western Russia, where clinical cases have not been reported, were marked as suitable for CCHFV circulation. The best outcome of the joint use of chorotypes of both ticks and vertebrates produced indexes of reliability similar to the previous approaches, with a slightly higher value of threshold, meaning for a somewhat worse performing model. The output of chorotypes is similar projections made from climate-only models. While it is interesting that summarized layers of information on ticks and vertebrates can produce a better outcome than climate only, the result is not yet finely tuned.

### 4). The best model for CCHFV occurrence uses human-biting ticks, vertebrate chorotypes, climate variables and livestock density

**Fig 10:**
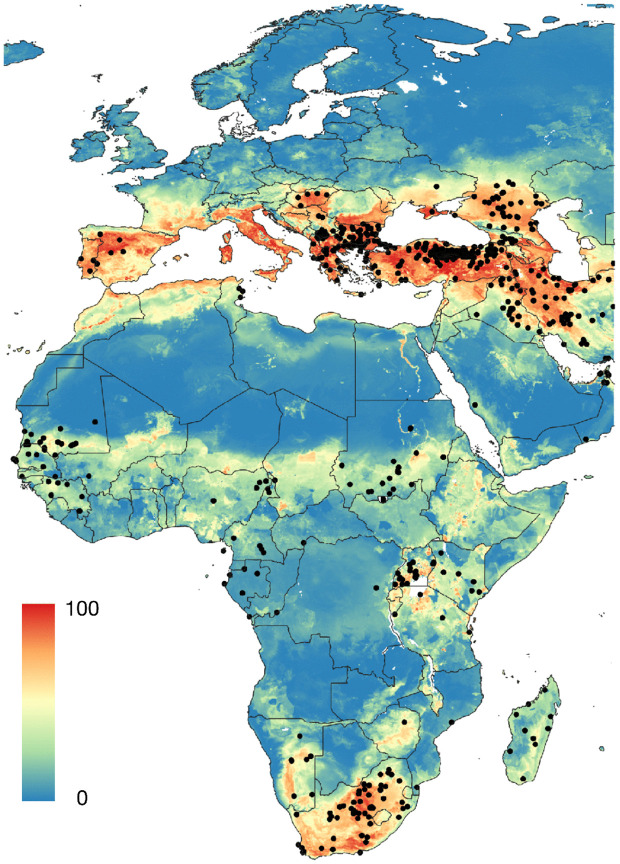
Probability of occurrence of CCHFV calculated with the distribution of human-biting ticks, the chorotypes of wild vertebrates, and the density of livestock (cattle, sheep, goats). The species of human-biting ticks are *Amblyomma hebraeum, Amblyomma variegatum, Dermacentor marginatus, Dermacentor reticulatus, Hyalomma aegyptium, Hyalomma anatolicum, Hyalomma excavatum, Hyalomma marginatum, Hyalomma rufipes, Hyalomma scupense, Hyalomma truncatum, Hyalomma turanicum,* and *Ixodes ricinus*. The base layer of the maps was downloaded from https://www.naturalearthdata.com/downloads/50m-cultural-vectors/ and modified (raster and additional vectors) in qGIS.

We aimed to produce a total evidence model, using explanatory variables of environmental features (climate) and a combined framework incorporating human-biting ticks, vertebrate chorotypes, and livestock density (as a proxy for human–livestock contact). The best-performing models consistently used either MARS or MaxEnt with the combined explanatory set (Fig 10), producing the highest sensitivity/specificity balances, lower omission rates, and higher kappa scores. Model performance metrics are summarised in Table 2.

**Table 2:** Summary of the outcome of algorithms modelling the distribution of CCHFV, with combinations of different explanatory variables. All the combinations included the nine variable species, that were also entered alone, as a final demonstration of the power of climate to discriminate the sites of CCHFV. “HBT+Livestock” refers to the joint use of human-biting ticks, chorotypes of vertebrates, and the density of livestock. “Complete chorotypes” refers to the simultaneous use of the chorotypes of both ticks and vertebrates. Threshold indicates the optimal value to convert the habitat suitability map into a binary presence/absence map (higher value is a better model).

Combination of variables and algorithm	Threshold	AUC	Omission rate	Sensitivity	Specificity	Accuracy
Only climate GAM	0.2420	0.6619	0.3170	0.6825	0.6833	0.6830
Only climate MARS	0.2010	0.6681	0.2901	0.5917	0.6887	0.6899
Only climate SVM	0.3201	0.6495	0.3057	0.6834	0.6853	0.6843
Only climate MaxEnt	0.4283	0.8598	0.2142	0.7859	0.7858	0.7858
Only ticks GAM	0.4240	0.8753	0.1903	0.8083	0.8107	0.8097
Only ticks MARS	0.3860	0.8803	0.1907	0.8083	0.8100	0.8093
Only ticks SVM	0.3740	0.8680	0.2037	0.7954	0.7972	0.7963
Only ticks	0.3947	0.8745	0.1949	0.8040	0.8060	0.8051
Chorotypes of ticks GAM	0.4240	0.8753	0.1903	0.8083	0.8107	0.8097
Chorotypes of ticks MARS	0.3860	0.8803	0.1907	0.8083	0.8100	0.8093
Chorotypes of ticks SVM	0.3740	0.8680	0.2037	0.7954	0.7972	0.7963
Chorotypes of ticks MaxEnt	0.3947	0.8745	0.1949	0.8040	0.8060	0.8051
Chorotypes of vertebrates GAM	0.4900	0.8700	0.2108	0.7899	0.7887	0.7892
Chorotypes of vertebrates MARS	0.4240	0.8900	0.1899	0.8120	0.8087	0.8101
Chorotypes of vertebrates SVM	0.4080	0.8669	0.1986	0.8028	0.8000	0.8014
Chorotypes of vertebrates MaxEnt	0.4383	0.8846	0.1876	0.8123	0.8125	0.8124
Complete chorotypes GAM	0.4360	0.8623	0.2151	0.7853	0.7847	0.7849
Complete chrotypes MARS	0.3970	0.8758	0.2039	0.7954	0.7967	0.7961
Complete chorotypes SVM	0.4350	0.8492	0.2152	0.7853	0.7843	0.7848
Complete chorotypes MaxEnt	0.4227	0.8625	0.2114	0.7886	0.7886	0.7886
HBT+Livestock GAM	0.4860	0.9080	0.1736	0.8262	0.8266	0.8264
HBT+Livestock MARS	0.4480	0.9129	0.1569	0.8421	0.8438	0.8431
HBT+Livestock SVM	0.5050	0.8903	0.1733	0.8262	0.8272	0.8267
HBT+Livestock MaxEnt	0.4797	0.9038	0.1680	0.8315	0.8325	0.8320

Overall, our results indicate that the spatial distribution of CCHFV is best explained by a combination of (i) the probability of occurrence of human-biting ticks, (ii) vertebrate community composition (i.e. the chorotypes of vertebrates), (iii) livestock density, and (iv) climate covariates. This multidimensional approach outperformed models developed on climate-only, tick-only, and combinations of explanatory variables, reflecting the multifactorial nature of CCHFV transmission and highlighting the importance of integrating host–vector–environment interactions in large-scale epidemiological models.

## Discussion

### 1. Limitations of climate-based models and the need for biotic context

This study presents the most comprehensive analysis to date, integrating abiotic and biotic determinants of the epidemiology of Crimean–Congo haemorrhagic fever by forecasting the potential distribution of CCHFV. By compiling reports of human cases, virus isolation sites, and molecular and serological datasets from both humans and animals, we generated and made publicly available a spatially explicit dataset spanning from northern Europe to southern Africa, and from the Atlantic to mid-Asia. Data from central and eastern Russia were intentionally excluded due to inconsistencies in reporting.

Our results challenge the assumption that CCHFV occupies a distinct climatic niche. Models based solely on climatic covariates yielded biased and overly extensive predictions that failed to align with observed occurrences. Climate-only models consistently overestimated northern suitability, a pattern previously noted in studies projecting CCHFV risk in Europe. Such models often identified regions lacking established populations of competent tick vectors and without records of human or animal infection [15,16,18,45]. Consequently, climate-based models alone appear inadequate as proxies for CCHFV epidemiology, as they tend to produce artifactual high-risk areas across central and northern Europe where only sporadic, imported ticks have been detected with no evidence of establishment [46,47]. To note, the records of the virus have been entered without information about the virus lineage [13] for model training. This is a topic worthy of research since our results on Multidimensional Scaling showed an unusual number of clusters within the raw records of the virus.

Previous research has largely focused on climate-derived variables as primary determinants of tick distributions and, by extension, CCHFV risk. This emphasis arises from the dual role of ticks as both vectors and reservoirs of the virus [48]. Yet, a wide and poorly characterised range of vertebrate hosts may also act as infection amplifiers [4,19]. While climate undoubtedly influences tick ecology, recent studies have demonstrated the added importance of landscape and biotic (vertebrate and tick-derived) factors in explaining large-scale CCHFV distribution [14,49].

Models using only tick distributions as predictors were similarly limited. Although a RF algorithm identified the likely combination of tick species involved in viral circulation, models relying exclusively on tick presence under-predicted the known range of CCHFV. This limitation likely reflects the complexity of co-occurring tick and vertebrate interactions, probably together with the genetic variability of the virus, which obscures individual species’ predictive value. It remains uncertain whether these interactions, or the absence of climatic information, contributed most to model instability. Future modelling efforts should explore these relationships using simplified and interpretable frameworks.

### 2. An approach Driven by Host–Vector Interactions

The most robust predictions were obtained when integrating distributions of human-biting ticks, livestock density, and host chorotypes, together with climate. This combination accurately captured approximately 91% of known CCHFV occurrences. Vertebrate chorotypes [37,39] enhanced predictive performance by identifying host assemblages most likely to sustain viral amplification. Notably, no single chorotype is uniquely associated with the virus; rather, each chorotype’s range aligns spatially with parts of the known CCHFV distribution. These biotic predictors reduced model dimensionality (Reulen & Kneib, 2016) while improving the delineation of both northern and African range limits. Vertebrates likely maintain viral circulation by supporting high tick densities and prolonged viraemia in medium-to large-sized hosts, while immature ticks feeding on small mammals may facilitate local persistence [50]. Incorporating vertebrate community structure therefore bridges vector ecology and pathogen transmission, yielding more ecologically realistic forecasts.

Our findings support an ecological generalist model of CCHFV transmission, as proposed [6]. Under this framework, the virus is not restricted to a narrow set of tick species. Instead, primary human-biting vectors sustain transmission to humans throughout most endemic regions, while secondary tick species maintain circulation among amplifying hosts, predominantly Artiodactyla [27]. These ticks typically rely on Rodentia, Lagomorpha and Insectivora as maintenance hosts for immature stages [20].

Taken together, the presence of CCHFV appears to be a matter of density: more competent tick vectors, more vertebrates capable of sustaining immature ticks, and more hosts that can amplify infection. Human transmission would thus arise from close contact with tick-infested habitats. This system explains the broad geographical extent of human cases without invoking strict molecular coevolution between ticks and vertebrates—an assumption not yet empirically tested.

A variable set of species is likely responsible for CCHFV transmission across different regions. *Hyalomma* spp. consistently emerge as key vectors, together with several *Rhipicephalus* spp. In Europe, transmission is primarily associated with *H. marginatum*, *H. scupense*, and *H. aegyptium*. The role of *D. marginatus* in virus circulation remains unclear, given its partial range overlap with *H. marginatum*; there are no *bona fide* records of CCHFV in *D. marginatus*, as detections refer only to feeding specimens [51]. Our study also provides indirect evidence regarding the potential involvement of *I. ricinus*, long listed among candidate vectors [6]. Engorging ticks, of any species feeding on an infectious animal still may contribute to the infection of humans when those ticks are removed or crushed. The northern fringe of predicted suitability coincides with overlap between *I. ricinus* and *H. marginatum*, the latter being the principal human-biting vector in the Mediterranean region. We hypothesise that *I. ricinus* may act as a vector on rare occasions where ranges overlap, *I. ricinus* being a secondary vector unable to sustain the infection in the absence of primary tic vectors like *Hyalomma* spp., although this remains unconfirmed. Laboratory evidence of viral replication in *I. ricinus* cell lines [52] and detections in questing ticks in Turkey [53] warrant further experimental validation.

In central Africa and the Sahel, the combination of human-biting tick presence and livestock density delineated potential transmission hotspots, possibly linked to peri-domestic slaughter or bushmeat practices that increase human exposure. Our approach also identified plausible risk zones in under-studied regions such as Madagascar, where the distribution of *A. variegatum*, the island’s only human-biting tick, matches reports of CCHFV circulation [54]. Overall, our results indicate a widespread viral range, efficiently maintained by ticks feeding on Artiodactyla.

Our best model also highlighted areas in central Africa that may be unsuitable for CCHFV, where ruminant-associated ticks do not overlap with human-biting species, supporting the observed absence of human cases or serological evidence. A further point of uncertainty concerns species such as *R. rossicus* and *R. decoloratus*, which emerged as positively associated with human transmission. The virus has been detected in both species [51], both bite humans [27] and both inhabit regions where human cases occur. At least *R. decoloratus* relies heavily on cattle, suggesting potential for substantial viral circulation. However, these species also overlap with well-established vectors such as *Hyalomma* spp., underscoring the need to clarify their vectorial competence.

The hypothesis of generalist ecological associations contrasts with evidence suggesting potential molecular links between viral lineages and specific tick taxa [1,55]. Our findings indicate spatial independence between the ranges of major tick vectors in the Western Palaearctic and the Afrotropics. If CCHFV lineages were tightly adapted to particular tick species, cross-regional spread would require genetic adaptation. However, reports of viral strains emerging in unexpected regions support our modelling results and reinforce the view of CCHFV as an ecologically flexible system, potentially facilitated by animal trade and migratory birds [56,57].

Despite its scope, this study has limitations. Data scarcity across central and eastern Asia hindered comprehensive modelling at the scale of the entire Palaearctic. Furthermore, the spatial resolution of serological data, often aggregated to administrative units, may have introduced minor inaccuracies. Large-scale datasets from central Asia and Africa [58,59] were excluded due to coarse reporting that precluded consistent spatial matching. Nonetheless, the integration of human and animal serology, human-biting tick distributions, and vertebrate chorotypes produced robust and ecologically coherent predictions, emphasising the value of combining biotic and abiotic dimensions in future epidemiological frameworks.

## Conclusions

Accurate mapping of CCHFV distribution requires a multifactorial approach that integrates both abiotic and biotic predictors. Models based solely on climate or on the ranges of known tick vectors generate biased outputs. Our findings indicate that CCHFV lacks a strict climatic niche and that its persistence depends on the co-occurrence of vector and vertebrate communities. Incorporating human-biting tick distributions, livestock density, and vertebrate chorotypes significantly improved predictive performance, providing a mechanistic understanding of the ecological processes sustaining virus circulation. This integrative framework establishes a robust foundation for forecasting CCHFV risk and highlights the importance of combining high-resolution ecological, vector, and host data with environmental variables in future studies of vector-borne zoonoses.

## Supporting information

S1 Table The species of ticks included in this study. Notes about its importance as human biting ticks have been included using the same terms as in the original compilation (Guglielmone and Robbins, 2018). Parasitism on Artiodactyla is included since medium and large ungulates are considered important vertebrates contributing to amplification of CCHFV. Categories of the prevalence of ticks on these groups of hosts are identical to the original report. Ticks belonging to the genus *Haemaphysalis* were originally included in the study but later removed because the lack of association with the records of CCHFV.
(DOCX)

S2 Table The compilation of serological surveys from literature. The file is organized in four sheets of information. It includes one sheet for data compiled from humans, another sheet for data generated from animals, and a third one that includes the rejected papers in the bibliographical query and revision. A four sheet defines the meaning of the columns of the data on humans or animals.
(XLSX)

S3 Table An ODMAP checklist for models on ticks. The file follow the protocols mentioned by Zurell et al. [60] regarding the modelling carried out on the climate suitability for the ticks used later as predictive layers of CCHFV.
(RTF)

S4 Table An ODMAP checklist for models on vertebrates. The file follow the protocols mentioned by Zurell et al. [60] regarding the modelling carried out on the climate suitability for the vertebrates used later as predictive layers of CCHFV.
(RTF)

S5 Table An ODMAP checklist for models of CCHFV. The file follow the protocols mentioned by Zurell et al. [60] regarding the modelling carried out on the range CCHFV.
(RTF)

S1 Fig The representation in the reduced space of the chorotypes of the ticks species included in this study. Dots are placed along the coordinates of chorotypes 1 and 2, with colour explaining the ordination in the chorotype 3. The chart intends to be informative but not exhaustive as some points are very near in the reduced space and their labels could not be separated.
(PNG)

S2 Fig The representation in the reduced space of the chorotypes of the genera of vertebrates included in this study. Dots are placed along the coordinates of chorotypes 1 and 2, with colour explaining the ordination in the chorotype 3. The chart intends to be informative but not exhaustive as some points are near in the reduced space and their labels could not be separated.
(PNG)

## References

[1] Papa A, Marklewitz M, Paraskevopoulou S, Garrison AR, Alkhovsky SV, Avšič-Županc T, et al. History and classification of Aigai virus (formerly Crimean-Congo haemorrhagic fever virus genotype VI). J Gen Virol. 2022;103(4):001734. doi: 10.1099/jgv.0.001734 35412967 PMC10026732

[2] Hawman DW, Feldmann H. Crimean-Congo haemorrhagic fever virus. Nat Rev Microbiol. 2023;21(7):463–77. doi: 10.1038/s41579-023-00871-9 36918725 PMC10013989

[3] Shepherd AJ, Swanepoel R, Shepherd SP, Leman PA, Mathee O. Viraemic transmission of Crimean-Congo haemorrhagic fever virus to ticks. Epidemiol Infect. 1991;106(2):373–82. doi: 10.1017/s0950268800048524 1902186 PMC2272004

[4] Spengler JR, Estrada-Peña A, Garrison AR, Schmaljohn C, Spiropoulou CF, Bergeron É, et al. A chronological review of experimental infection studies of the role of wild animals and livestock in the maintenance and transmission of Crimean-Congo hemorrhagic fever virus. Antiviral Res. 2016;135:31–47. doi: 10.1016/j.antiviral.2016.09.013 27713073 PMC5102700

[5] Gordon SW, Linthicum KJ, Moulton JR. Transmission of Crimean-Congo hemorrhagic fever virus in two species of Hyalomma ticks from infected adults to cofeeding immature forms. Am J Trop Med Hyg. 1993;48(4):576–80. doi: 10.4269/ajtmh.1993.48.576 8480867

[6] Hoogstraal H. The epidemiology of tick-borne Crimean-Congo hemorrhagic fever in Asia, Europe, and Africa. J Med Entomol. 1979;15(4):307–417. doi: 10.1093/jmedent/15.4.307 113533

[7] Gale P, Stephenson B, Brouwer A, Martinez M, de la Torre A, Bosch J, et al. Impact of climate change on risk of incursion of Crimean-Congo haemorrhagic fever virus in livestock in Europe through migratory birds. J Appl Microbiol. 2012;112(2):246–57. doi: 10.1111/j.1365-2672.2011.05203.x 22118269

[8] Gargili A, Estrada-Peña A, Spengler JR, Lukashev A, Nuttall PA, Bente DA. The role of ticks in the maintenance and transmission of Crimean-Congo hemorrhagic fever virus: a review of published field and laboratory studies. Antiviral Res. 2017;144:93–119. doi: 10.1016/j.antiviral.2017.05.010 28579441 PMC6047067

[9] Celina SS, Italiya J, Tekkara AO, Černý J. Crimean-Congo haemorrhagic fever virus in ticks, domestic, and wild animals. Front Vet Sci. 2025;11:1513123. doi: 10.3389/fvets.2024.1513123 39897158 PMC11782920

[10] Durden LA, Logan TM, Wilson ML, Linthicum KJ. Experimental vector incompetence of a soft tick, Ornithodoros sonrai (Acari: Argasidae), for Crimean-Congo hemorrhagic fever virus. J Med Entomol. 1993;30(2):493–6. doi: 10.1093/jmedent/30.2.493 8459431

[11] Estrada-Peña A, Cevidanes A, Sprong H, Millán J. Pitfalls in tick and tick-borne pathogens research, some recommendations and a call for data sharing. Pathogens. 2021;10(6):712. doi: 10.3390/pathogens10060712 34200175 PMC8229135

[12] Belobo JTE, Kenmoe S, Kengne-Nde C, Emoh CPD, Bowo-Ngandji A, Tchatchouang S, et al. Worldwide epidemiology of Crimean-Congo Hemorrhagic Fever Virus in humans, ticks and other animal species, a systematic review and meta-analysis. PLoS Negl Trop Dis. 2021;15(4):e0009299. doi: 10.1371/journal.pntd.0009299 33886556 PMC8096040

[13] Gruber CEM, Bartolini B, Castilletti C, Mirazimi A, Hewson R, Christova I, et al. Geographical variability affects CCHFV detection by RT-PCR: a tool for in-silico evaluation of molecular assays. Viruses. 2019;11(10):953. doi: 10.3390/v11100953 31623214 PMC6833031

[14] Ilboudo AK, Oloo SO, Sircely J, Nijhof AM, Bett B. Spatial analysis and risk mapping of Crimean-Congo hemorrhagic fever (CCHF) in Sub-saharan Africa. Sci Rep. 2025;15(1):2292. doi: 10.1038/s41598-025-85873-8 39825034 PMC11742035

[15] Messina JP, Pigott DM, Golding N, Duda KA, Brownstein JS, Weiss DJ, et al. The global distribution of Crimean-Congo hemorrhagic fever. Trans R Soc Trop Med Hyg. 2015;109(8):503–13. doi: 10.1093/trstmh/trv050 26142451 PMC4501401

[16] Messina JP, Wint GRW. The spatial distribution of crimean-congo haemorrhagic fever and its potential vectors in Europe and beyond. Insects. 2023;14(9):771. doi: 10.3390/insects14090771 37754739 PMC10532370

[17] Okely M, Anan R, Gad-Allah S, Samy AM. Mapping the environmental suitability of etiological agent and tick vectors of Crimean-Congo hemorrhagic fever. Acta Trop. 2020;203:105319. doi: 10.1016/j.actatropica.2019.105319 31874130

[18] Celina SS, Černý J, Samy AM. Mapping the potential distribution of the principal vector of Crimean-Congo haemorrhagic fever virus Hyalomma marginatum in the Old World. PLoS Negl Trop Dis. 2023;17(11):e0010855. doi: 10.1371/journal.pntd.0010855 38011221 PMC10703407

[19] Spengler JR, Bergeron É, Rollin PE. Seroepidemiological studies of crimean-congo hemorrhagic fever virus in domestic and wild animals. PLoS Negl Trop Dis. 2016;10(1):e0004210. doi: 10.1371/journal.pntd.0004210 26741652 PMC4704823

[20] Spengler JR, Estrada-Peña A. Host preferences support the prominent role of Hyalomma ticks in the ecology of Crimean-Congo hemorrhagic fever. PLoS Negl Trop Dis. 2018;12(2):e0006248. doi: 10.1371/journal.pntd.0006248 29420542 PMC5821391

[21] Cumming GS. Host distributions do not limit the species ranges of most African ticks (Acari: Ixodida). Bull Ent Res. 1999;89:303–27.

[22] Estrada-Peña A, de la Fuente J. Machine learning algorithms for the evaluation of risk by tick-borne pathogens in Europe. Ann Med. 2024;56(1):2405074. doi: 10.1080/07853890.2024.2405074 39348264 PMC11443563

[23] Rubel F, Brugger K, Belova OA, Kholodilov IS, Didyk YM, Kurzrock L, Kahl O. Vectors of disease at the northern distribution limit of the genus Dermacentor in Eurasia: D. reticulatus and D. silvarum. Exp. Appl. Acarol. 2020; 82, 95–123. 32815071 10.1007/s10493-020-00533-y PMC7471206

[24] Rubel F, Brugger K, Chitimia-Dobler L, Dautel H, Meyer-Kayser E, Kahl O. Atlas of ticks (Acari: Argasidae, Ixodidae) in Germany. Exp Appl Acarol. 2021;84(1):183–214. doi: 10.1007/s10493-021-00619-1 33939100 PMC8102463

[25] Rubel F, Brugger K, Pfeffer M, Chitimia-Dobler L, Didyk YM, Leverenz S, et al. Geographical distribution of Dermacentor marginatus and Dermacentor reticulatus in Europe. Ticks Tick Borne Dis. 2016;7(1):224–33. doi: 10.1016/j.ttbdis.2015.10.015 26552893

[26] Global Biodiversity Information Facility (GBIF) downloads. DOI10.15468/dl.6bu4cv, Sep 11th, 2023; DOI10.15468/dl.eha28h, Dec 23th, 2023; DOI10.15468/dl.6e4kgm, May 14th, 2024; DOI10.15468/dl.3qjbcq, May 15th, 2024; DOI10.15468/dl.d7wjqn, May 15th, 2024; DOI10.15468/dl.syj5y7, May 15th, 2024; DOI10.15468/dl.pr5wuh, May 18th, 2024; DOI10.15468/dl.wmaa9j, May 18th, 2024; DOI10.15468/dl.ggy6uc, May 18th, 2024; DOI10.15468/dl.w5tbav, May 18th, 2024; DOI10.15468/dl.eyupng, May 18th, 2024; DOI10.15468/dl.32srrz, May 19th, 2024; DOI10.15468/dl.wkak83, May 19th, 2024; DOI10.15468/dl.9dhvkm, May 19th, 2024; DOI10.15468/dl.7qfhzu, May 19th, 2024; DOI10.15468/dl.hnj5uj, May 20th, 2024; DOI10.15468/dl.ckfsyd, May 20th, 2024; DOI10.15468/dl.tk47bc, May 20th, 2024; DOI10.15468/dl.n4cncx, May 20th, 2024; DOI10.15468/dl.dqumac, May 20th, 2024; DOI10.15468/dl.mnppbq, May 20th, 2024; DOI10.15468/dl.7vxe4x, May 20th, 2024; DOI10.15468/dl.yaj3vd, May 21th, 2024; DOI10.15468/dl.pnu5rj, May 21th, 2024; DOI10.15468/dl.nnmvcd, May 21th, 2024; DOI10.15468/dl.7587hj, May 21th, 2024; DOI10.15468/dl.pr39ws, May 21th, 2024; DOI10.15468/dl.qjxwnv, May 21th, 2024; DOI10.15468/dl.96n8yy, Aug 12th, 2024; DOI10.15468/dl.nj9uv7, Aug 12th, 2024; DOI10.15468/dl.d92vcx, Aug 12th, 2024; DOI10.15468/dl.akxg7k, Aug 12th, 2024; DOI10.15468/dl.m56tgg, Aug 12th, 2024; DOI10.15468/dl.jxcnr2, Aug 12th, 2024; DOI10.15468/dl.mkcrmh, Aug 12th, 2024; DOI10.15468/dl.2ugeey, Aug 12th, 2024; DOI10.15468/dl.sex3gk, Aug 16th, 2024; DOI10.15468/dl.ubct8s, Aug 16th, 2024; DOI10.15468/dl.vjtued, Aug 16th, 2024; DOI10.15468/dl.grk2fy, Aug 16th, 2024; DOI10.15468/dl.kdz63g, Aug 12th, 2024; DOI10.15468/dl.vgugv6, Aug 20th, 2024; DOI10.15468/dl.fn8z8t, Aug 20th, 2024; DOI10.15468/dl.esmypu, Aug 20th, 2024; DOI10.15468/dl.rz7mzh, Aug 20th, 2024; DOI10.15468/dl.p66q7s, Aug 20th, 2024; DOI10.15468/dl.38dgud, Aug 20th, 2024; DOI10.15468/dl.wdsnwb, Aug 20th, 2024; DOI10.15468/dl.g586yx, Aug 20th, 2024; DOI10.15468/dl.ukbmrp, Aug 20th, 2024; DOI10.15468/dl.zzevfw, Aug 20th, 2024; DOI10.15468/dl.mrpdsx, Aug 20th, 2024; DOI10.15468/dl.3jtcxt, Aug 20th, 2024; DOI10.15468/dl.h5z7j4, Aug 20th, 2024; DOI10.15468/dl.znekcc, Aug 20th, 2024; DOI10.15468/dl.vux27w, Aug 20th, 2024; DOI10.15468/dl.sw2apb, Aug 20th, 2024; DOI10.15468/dl.a94rdk, Oct 19th, 2024; DOI10.15468/dl.wurvz7. 2024.

[27] Guglielmone AA, Robbins RG, Apanaskevich DA, Petney TN, Estrada-Peña A, Horak IG. The hard ticks of the world. Dordrecht: Springer; 2014.

[28] Abatzoglou JT, Dobrowski SZ, Parks SA, Hegewisch KC. TerraClimate, a high-resolution global dataset of monthly climate and climatic water balance from 1958-2015. Sci Data. 2018;5:170191. doi: 10.1038/sdata.2017.191 29313841 PMC5759372

[29] Hijmans R. Terra: spatial data analysis. 2025.

[30] Scharlemann JPW, Benz D, Hay SI, Purse BV, Tatem AJ, Wint GRW, et al. Global data for ecology and epidemiology: a novel algorithm for temporal Fourier processing MODIS data. PLoS One. 2008;3(1):e1408. doi: 10.1371/journal.pone.0001408 18183289 PMC2171368

[31] Estrada-Peña A, Alexander N, Wint GRW. Perspectives on modelling the distribution of ticks for large areas: so far so good?. Parasit Vectors. 2016;9:179. doi: 10.1186/s13071-016-1474-9 27030357 PMC4815247

[32] Schmitt S, Pouteau R, Justeau D, de Boissieu F, Birnbaum P. ssdm: an r package to predict distribution of species richness and composition based on stacked species distribution models. Methods Ecol Evol. 2017;8(12):1795–803. doi: 10.1111/2041-210x.12841

[33] Kass JM, Pinilla‐Buitrago GE, Paz A, Johnson BA, Grisales‐Betancur V, Meenan SI, et al. wallace 2: a shiny app for modeling species niches and distributions redesigned to facilitate expansion via module contributions. Ecography. 2023;2023(3). doi: 10.1111/ecog.06547

[34] Elith J, Leathwick JR. Species distribution models: ecological explanation and prediction across space and time. Annu Rev Ecol Evol Syst. 2009;40:677–97. doi: 10.1146/annurev.ecolsys.110308.120159

[35] Merow C, Smith MJ, Silander JA Jr. A practical guide to MaxEnt for modeling species’ distributions: what it does, and why inputs and settings matter. Ecography. 2013;36(10):1058–69. doi: 10.1111/j.1600-0587.2013.07872.x

[36] Warren DL, Seifert SN. Ecological niche modeling in Maxent: the importance of model complexity and the performance of model selection criteria. Ecol Appl. 2011;21(2):335–42. doi: 10.1890/10-1171.1 21563566

[37] Real R, Márquez AL, Estrada A, Muñoz AR, Vargas JM. Modelling chorotypes of invasive vertebrates in mainland Spain. Divers Distrib. 2008;14:364–73.

[38] Estrada-Peña A, Wijburg SR, Sprong H. Climate driven patterns shape clusters of co-occurring ticks and vertebrates in the Western Palearctic-Tropics. Int J Parasitol. 2026;56(3):104735. doi: 10.1016/j.ijpara.2025.09.005 40983158

[39] Dapporto L, Cini A, Vodă R, Dincă V, Wiemers M, Menchetti M, et al. Integrating three comprehensive data sets shows that mitochondrial DNA variation is linked to species traits and paleogeographic events in European butterflies. Mol Ecol Resour. 2019;19(6):1623–36. doi: 10.1111/1755-0998.13059 31325412

[40] Dapporto L, Ramazzotti M, Fattorini S, Talavera G, Vila R, Dennis RLH. recluster: an unbiased clustering procedure for beta‐diversity turnover. Ecography. 2013;36(10):1070–5. doi: 10.1111/j.1600-0587.2013.00444.x

[41] Estrada-Peña A, Zatansever Z, Gargili A, Aktas M, Uzun R, Ergonul O, et al. Modeling the spatial distribution of crimean-congo hemorrhagic fever outbreaks in Turkey. Vector Borne Zoonotic Dis. 2007;7(4):667–78. doi: 10.1089/vbz.2007.0134 18047397

[42] Souza T. Non-metric Multidimensional Scaling (NMDS). Advanced statistical analysis for soil scientists. Cham: Springer; 2025.

[43] Breiman L. Random forests. Mach Learn. 2001;45:5–32. doi: 10.1023/A:1010933404324

[44] Guglielmone AA, Robbins RG. Hard ticks (Acari: Ixodida: Ixodidae) parasitizing humans. Cham: Springer; 2018.

[45] Leblebicioglu H, Ozaras R, Erciyas-Yavuz K. Emergence of Crimean-Congo hemorrhagic fever. Trans R Soc Trop Med Hyg. 2015;109(11):676–8. doi: 10.1093/trstmh/trv083 26464230

[46] McGinley L, Hansford KM, Cull B, Gillingham EL, Carter DP, Chamberlain JF, et al. First report of human exposure to Hyalomma marginatum in England: further evidence of a Hyalomma moulting event in north-western Europe?. Ticks Tick Borne Dis. 2021;12(1):101541. doi: 10.1016/j.ttbdis.2020.101541 33007668

[47] Uiterwijk M, Ibáñez-Justicia A, van de Vossenberg B, Jacobs F, Overgaauw P, Nijsse R, et al. Imported Hyalomma ticks in the Netherlands 2018-2020. Parasit Vectors. 2021;14(1):244. doi: 10.1186/s13071-021-04738-x 33962655 PMC8106226

[48] Turell MJ. Role of ticks in the transmission of Crimean-Congo hemorrhagic fever virus. In: Crimean-Congo hemorrhagic fever: a global perspective. Dordrecht: Springer; 2007. 143–54.

[49] Lule SA, Gibb R, Kizito D, Nakanjako G, Mutyaba J, Balinandi S, et al. Widespread exposure to Crimean-Congo haemorrhagic fever in Uganda might be driven by transmission from Rhipicephalus ticks: evidence from cross-sectional and modelling studies. J Infect. 2022;85(6):683–92. doi: 10.1016/j.jinf.2022.09.016 36152736

[50] Reulen H, Kneib T. Boosting multi-state models. Lifetime Data Anal. 2016;22(2):241–62. doi: 10.1007/s10985-015-9329-9 25990764

[51] S Celina S, Černý J. Genetic background of adaptation of Crimean-Congo haemorrhagic fever virus to the different tick hosts. PLoS One. 2024;19(4):e0302224. doi: 10.1371/journal.pone.0302224 38662658 PMC11045102

[52] Bell-Sakyi L, Kohl A, Bente DA, Fazakerley JK. Tick cell lines for study of Crimean-Congo hemorrhagic fever virus and other arboviruses. Vector Borne Zoonotic Dis. 2012;12(9):769–81. doi: 10.1089/vbz.2011.0766 21955214 PMC3438810

[53] Ahrabi SZ, Akyildiz G, Kar S, Keles AG. Detection of the Crimean-Congo Hemorrhagic fever virus genome in questing Ixodes spp. and Haemaphysalis spp. in the periurban forestry areas of Istanbul: has a new biorisk emerged?. Vector Borne Zoonotic Dis. 2023;23(10):528–36. doi: 10.1089/vbz.2023.0023 37527191

[54] Stachurski F. Variabilité de l’infestation de bovins par Amblyomma variegatum et son utilisation possible pour la lutte contre cette tique. Rev Elev Med Vet Pays Trop. 1993;46:341–8. 8134651

[55] Mild M, Simon M, Albert J, Mirazimi A. Towards an understanding of the migration of Crimean-Congo hemorrhagic fever virus. J Gen Virol. 2010;91(Pt 1):199–207. doi: 10.1099/vir.0.014878-0 19812264

[56] De Liberato C, Frontoso R, Magliano A, Montemaggiori A, Autorino GL, Sala M, et al. Monitoring for the possible introduction of Crimean-Congo haemorrhagic fever virus in Italy based on tick sampling on migratory birds and serological survey of sheep flocks. Prev Vet Med. 2018;149:47–52. doi: 10.1016/j.prevetmed.2017.10.014 29290300

[57] Mancuso E, Toma L, Polci A, d’Alessio SG, Di Luca M, Orsini M, et al. Crimean-Congo hemorrhagic fever virus genome in tick from migratory bird, Italy. Emerg Infect Dis. 2019;25(7):1418–20. doi: 10.3201/eid2507.181345 31211933 PMC6590740

[58] Fereidouni M, Apanaskevich DA, Pecor DB, Pshenichnaya NY, Abuova GN, Tishkova FH, et al. Crimean-Congo hemorrhagic fever virus in Central, Eastern, and South-eastern Asia. Virol Sin. 2023;38(2):171–83. doi: 10.1016/j.virs.2023.01.001 36669701 PMC10926685

[59] Temur AI, Kuhn JH, Pecor DB, Apanaskevich DA, Keshtkar-Jahromi M. Epidemiology of Crimean-Congo Hemorrhagic Fever (CCHF) in Africa-Underestimated for Decades. Am J Trop Med Hyg. 2021;104(6):1978–90. doi: 10.4269/ajtmh.20-1413 33900999 PMC8176481

[60] Zurell D, Franklin J, König C, Bouchet PJ, Dormann CF, Elith J, et al. A standard protocol for reporting species distribution models. Ecography. 2020;43(9):1261–77. doi: 10.1111/ecog.04960

