# Supplementary material for: Crimean–congo haemorrhagic fever virus circulates within broad ecological networks of ticks and vertebrates
Source: PLoS Negl Trop Dis. 2026 May 27;20(5):e0013783. doi: 10.1371/journal.pntd.0013783 (PMC13232941; doi:10.1371/journal.pntd.0013783)
Supplement: S1 Table — Notes about its importance as human biting ticks have been included using the same terms as in the original compilation (Guglielmone and Robbins, 2018). Parasitism on Artiodactyla is included since medium and large ungulates are considered important vertebrates contributing to amplification of CCHFV. Categories of the prevalence of ticks on these groups of hosts are identical to the original report. Ticks belonging to the genus Haemaphysalis were originally included in the study but later removed because the lack of association with the records of CCHFV. (DOCX) [file pntd.0013783.s001.docx]

Supplementary Table 1

| Species | Humans | Artiodactyla |
| --- | --- | --- |
| *Amblyomma astrion* |  | Very frequent |
| *Amblyomma cohaerens* |  | Slightly frequent |
| *Amblyomma compressum* |  | Slightly frequent |
| *Amblyomma eburneum* |  | Frequent |
| *Amblyomma exornatum* |  |  |
| *Amblyomma falsomarmoreum* |  | Frequent |
| *Amblyomma flavomaculatum* |  |  |
| *Amblyomma gemma* |  | Frequent |
| *Amblyomma hebraeum* | Frequent | Very frequent |
| *Amblyomma latum* |  |  |
| *Amblyomma lepidum* |  | Frequent |
| *Amblyomma marmoreum* |  | Very frequent |
| *Amblyomma nuttalli* |  |  |
| *Amblyomma paulopunctatum* |  | Frequent |
| *Amblyomma personatum* |  | Very frequent |
| *Amblyomma pomposum* |  | Frequent |
| *Amblyomma rhinocerotis* |  | Slightly frequent |
| *Amblyomma sparsum* |  |  |
| *Amblyomma splendidum* |  | Slightly frequent |
| *Amblyomma sylvaticum* |  |  |
| *Amblyomma tholloni* |  | Frequent |
| *Amblyomma variegatum* | Frequent | Very frequent |
| *Dermacentor circumguttatus* |  |  |
| *Dermacentor marginatus* | Very frequent | Slighty frequent |
| *Dermacentor reticulatus* | Frequent | Slighty frequent |
| *Hyalomma aegyptium* | Very frequent |  |
| *Hyalomma albiparmatum* |  |  |
| *Hyalomma anatolicum* | Frequent | Frequent |
| *Hyalomma asiaticum* |  |  |
| *Hyalomma dromedarii* |  | Very frequent |
| *Hyalomma excavatum* | Frequent | Frequent |
| *Hyalomma impeltatum* |  | Frequent |
| *Hyalomma impressum* |  | Frequent |
| *Hyalomma lusitanicum* |  | Very frequent |
| *Hyalomma marginatum* | Very frequent | Very frequent |
| *Hyalomma nitidum* |  |  |
| *Hyalomma rufipes* |  | Slightly frequent |
| *Hyalomma scupense* |  | Very frequent |
| *Hyalomma truncatum* | Frequent | Slightly frequent |
| *Hyalomma turanicum* | Frequent |  |
| *Ixodes persulcatus* | Very frequent | Frequent |
| *Ixodes pilosus* |  | Very frequent |
| *Ixodes rasus* |  | Relatively frequent |
| *Ixodes ricinus* | Very frequent | Very frequent |
| *Ixodes rubicundus* |  | Very frequent |
| *Ixodes trianguliceps* |  |  |
| *Ixodes ventalloi* |  |  |
| *Rhipicentor bicornis* |  |  |
| *Rhipicentor nuttalli* |  |  |
| *Rhipicephalus annulatus* |  | Very frequent |
| *Rhipicephalus appendiculatus* |  | Very frequent |
| *Rhipicephalus bequaerti* |  |  |
| *Rhipicephalus bergeoni* |  | Slightly frequent |
| *Rhipicephalus bursa* | Very frequent | Very frequent |
| *Rhipicephalus capensis* group |  | Slightly frequent |
| *Rhipicephalus capensis* |  | Slightly frequent |
| *Rhipicephalus complanatus* |  | Slightly frequent |
| *Rhipicephalus compositus* |  | Slightly frequent |
| *Rhipicephalus cuspidatus* |  | Slightly frequent |
| *Rhipicephalus decoloratus* |  | Very frequent |
| *Rhipicephalus distinctus* |  | Slightly frequent |
| *Rhipicephalus duttoni* |  | Frequent |
| *Rhipicephalus dux* |  |  |
| *Rhipicephalus evertsi group* |  | Very frequent |
| *Rhipicephalus exophthalmos* |  | Slightly frequent |
| *Rhipicephalus geigyi* |  | Frequent |
| *Rhipicephalus gertrudae group* |  | Slightly frequent |
| *Rhipicephalus glabroscutatum* |  | Slightly frequent |
| *Rhipicephalus guilhoni* |  | Frequent |
| *Rhipicephalus humeralis* |  | Slightly frequent |
| *Rhipicephalus interventus* |  | Frequent |
| *Rhipicephalus kochi* |  |  |
| *Rhipicephalus kohlsi* |  | Very frequent |
| *Rhipicephalus longiceps* |  | Slightly frequent |
| *Rhipicephalus longicoxatus* |  |  |
| *Rhipicephalus longus* |  |  |
| *Rhipicephalus lunulatus* |  |  |
| *Rhipicephalus maculatus* |  |  |
| *Rhipicephalus masseyi* |  |  |
| *Rhipicephalus microplus* |  | Very frequent |
| *Rhipicephalus muehlensi* |  |  |
| *Rhipicephalus muhsamae* |  | Frequent |
| *Rhipicephalus neumanni* |  | Frequent |
| *Rhipicephalus nitens* |  | Frequent |
| *Rhipicephalus oculatus* |  | Frequent |
| *Rhipicephalus planus* |  |  |
| *Rhipicephalus praetextatus* |  |  |
| *Rhipicephalus pravus* |  |  |
| *Rhipicephalus pulchellus* |  |  |
| *Rhipicephalus punctatus* |  |  |
| *Rhipicephalus pusillus* |  |  |
| *Rhipicephalus rossicus* |  | Slightly frequent |
| *Rhipicephalus sanguineus* group | Frequent |  |
| *Rhipicephalus senegalensis* |  | Slightly frequent |
| *Rhipicephalus simpsoni* |  |  |
| *Rhipicephalus simus* |  | Frequent |
| *Rhipicephalus sulcatus* |  | Slightly frequent |
| *Rhipicephalus supertritus* |  | Slightly frequent |
| *Rhipicephalus theileri* |  |  |
| *Rhipicephalus ziemani* |  |  |
