# Supplementary material for: Crimean–congo haemorrhagic fever virus circulates within broad ecological networks of ticks and vertebrates
Source: PLoS Negl Trop Dis. 2026 May 27;20(5):e0013783. doi: 10.1371/journal.pntd.0013783 (PMC13232941; doi:10.1371/journal.pntd.0013783)
Supplement: S3 Table — The file follow the protocols mentioned by Zurell et al. [60] regarding the modelling carried out on the climate suitability for the ticks used later as predictive layers of CCHFV. (RTF) [file pntd.0013783.s003.rtf]

ODMAP — vertebrates modelsO — OverviewStudy objectiveTo predict the geographic distribution of vertebrate genera across the Western Palearctic and Afrotropical regions in order to derive chorotypes (groups of co-occurring taxa), which are subsequently used as predictors in models of Crimean-Congo hemorrhagic fever virus distribution.Target taxaA comprehensive set of vertebrate genera (full list provided in Supplementary Material S1).Study areaWestern Palearctic and Afrotropics (72°N–36°S; 18°W–58°E).Temporal scopeContemporary occurrence records; environmental predictors represent current climatic conditions.Modelling objectiveEstimation of continuous habitat suitability for each genus.⸻ — DataOccurrence data	•	Source: Global Biodiversity Information Facility	•	Initial sample size: 8,114,309 records	•	Final sample size after filtering: 4,920,908 records	•	Coordinates: geographic (longitude, latitude)	•	Spatial thinning: applied at 4 km minimum distance to reduce spatial sampling bias	•	Response format: presence–pseudoabsence⸻seudoabsence design	•	Method: random sampling across the study area	•	Ratio: 10 pseudoabsences per presence (applied independently for each genus)	•	Spatial constraints: none	•	Consistency: identical procedure across all taxa and models⸻nvironmental predictors	•	Source: TerraClimate dataset	•	Variables:	•	Monthly maximum temperature	•	Monthly minimum temperature	•	Monthly vapour pressure deficit	•	Preprocessing:	•	Harmonic (Fourier) regression applied to monthly time series	•	First three coefficients retained per variable	•	Final predictor set: 9 variables	•	Spatial resolution: ~4 km	•	Collinearity:	•	Not explicitly filtered; Fourier coefficients are orthogonal by construction	•	Scaling: not applied⸻ — ModelAlgorithms	•	Random Forest	•	MARS (Multivariate Adaptive Regression Splines)	•	GAMM (Generalized Additive Mixed Models)	•	SVM (Support Vector Machines)	•	Maxent (implemented via maxnet)⸻mplementation	•	Framework: R (tidymodels for most algorithms; maxnet for Maxent)	•	Hyperparameters:	•	Default or internally tuned depending on algorithm	•	Maxent settings follow standard maxnet implementation (feature classes and regularization not explicitly tuned)	•	Data partitioning:	•	Random train/test split (70% training, 30% testing)⸻ — AssessmentEvaluation metrics	•	Area Under the ROC Curve (AUC)	•	True Skill Statistic (TSS)	•	Cohen’s Kappa	•	Accuracy	•	Calibration slope	•	Omission rate⸻hreshold selection	•	Threshold maximizing (sensitivity + specificity)⸻alidation strategy	•	Random hold-out validation (70/30 split)⸻ — Prediction	•	Output: continuous habitat suitability (0–1)	•	Spatial implementation: raster prediction using terra (SpatRaster)	•	Projection domain: current climatic conditions only	•	Extrapolation control: none applied⸻dditional note (critical for downstream use)Predicted suitability surfaces were subsequently used to derive vertebrate chorotypes, which were incorporated as predictor variables in downstream models of CCHFV distribution. These predictors are therefore model-derived and may carry propagated uncertainty.
