# Supplementary material for: Crimean–congo haemorrhagic fever virus circulates within broad ecological networks of ticks and vertebrates
Source: PLoS Negl Trop Dis. 2026 May 27;20(5):e0013783. doi: 10.1371/journal.pntd.0013783 (PMC13232941; doi:10.1371/journal.pntd.0013783)
Supplement: S4 Table — The file follow the protocols mentioned by Zurell et al. [60] regarding the modelling carried out on the climate suitability for the vertebrates used later as predictive layers of CCHFV. (RTF) [file pntd.0013783.s004.rtf]

ODMAP 2 — Tick modelsO — OverviewStudy objectiveTo predict the geographic distribution of tick species across the Western Palearctic and Afrotropical regions in order to derive chorotypes (groups of co-occurring species), later used as predictors in models of Crimean-Congo hemorrhagic fever virus distribution.Target taxaA comprehensive set of tick species across multiple genera (e.g., Amblyomma, Hyalomma, Rhipicephalus, Ixodes; full list provided in Supplementary Material S2).Study areaWestern Palearctic and Afrotropics (72°N–36°S; 18°W–58°E).Temporal scopeContemporary occurrence records; environmental predictors represent current climatic conditions.Modelling objectiveEstimation of continuous habitat suitability for each species.⸻ — DataOccurrence data	•	Source: Global Biodiversity Information Facility	•	Initial sample size: 92,404 records	•	Final sample size after filtering: 92,404 records (the dataset was cleaned previously)	•	Coordinates: geographic (longitude, latitude)	•	Spatial thinning: applied at 4 km minimum distance	•	Response format: presence–pseudoabsence⸻seudoabsence design	•	Method: random sampling across the study area	•	Ratio: 10 pseudoabsences per presence (applied independently per species)	•	Spatial constraints: none	•	Consistency: identical across all models⸻nvironmental predictors	•	Source: TerraClimate dataset	•	Variables:	•	Monthly maximum temperature	•	Monthly minimum temperature	•	Monthly vapour pressure deficit	•	Preprocessing:	•	Harmonic (Fourier) regression	•	First three coefficients retained per variable	•	Total predictors: 9	•	Spatial resolution: ~4 km	•	Collinearity:	•	Not addressed explicitly; predictors assumed orthogonal	•	Scaling: not applied⸻ — ModelAlgorithms	•	Random Forest	•	MARS	•	GAMM	•	SVM	•	Maxent (via maxnet)⸻mplementation	•	Framework: R (tidymodels, maxnet)	•	Hyperparameters:	•	Default or internally tuned depending on algorithm	•	Maxent settings follow standard maxnet defaults	•	Data partitioning:	•	Random train/test split (70% / 30%)⸻ — AssessmentEvaluation metrics	•	AUC	•	TSS	•	Kappa	•	Accuracy	•	Calibration slope	•	Omission rate⸻hreshold selection	•	Threshold maximizing (sensitivity + specificity)⸻alidation strategy	•	Random hold-out validation⸻ — Prediction	•	Output: continuous suitability (0–1)	•	Raster prediction: terra (SpatRaster)	•	Projection: current climate only	•	Extrapolation control: none⸻dditional note (critical for downstream use)Predicted suitability maps were used to derive tick chorotypes, which were subsequently incorporated as predictor variables in models of CCHFV distribution. As such, these predictors represent model-derived estimates and may introduce propagated uncertainty into downstream analyses.
