# Supplementary material for: Crimean–congo haemorrhagic fever virus circulates within broad ecological networks of ticks and vertebrates
Source: PLoS Negl Trop Dis. 2026 May 27;20(5):e0013783. doi: 10.1371/journal.pntd.0013783 (PMC13232941; doi:10.1371/journal.pntd.0013783)
Supplement: S5 Table — The file follow the protocols mentioned by Zurell et al. [60] regarding the modelling carried out on the range CCHFV. (RTF) [file pntd.0013783.s005.rtf]

ODMAP — CCHFV Distribution ModellingO — OverviewStudy objectiveTo predict the spatial distribution of Crimean–Congo haemorrhagic fever virus (CCHFV) across the Western Palearctic and Afrotropical regions using climate variables and modelled distributions of ticks and vertebrate hosts.Target taxonOrthonairovirus haemorrhagiae (Crimean–Congo haemorrhagic fever virus)Study area	•	Geographic extent: Western Palearctic and Afrotropics (72°N to 36°S; 18°W to 58°E)	•	Temporal scope: Current climatic conditions	•	Main modelling goal: To estimate the spatial distribution of CCHFV based on climatic suitability and the modelled distributions of vector ticks and vertebrate hosts⸻ — DataOccurrence data	•	Sources: Global Biodiversity Information Facility (GBIF) and published literature(including virus detections, human clinical cases, and animal/human serological records)	•	Initial sample size: 725	•	Final sample size after filtering: 725	•	Coordinate format: Longitude, Latitude	•	Spatial thinning: Not applied	•	Response variable: Presence with pseudoabsence generation⸻seudoabsence / background design	•	Method: Random sampling	•	Number of pseudoabsences per presence: 1,217	•	Spatial constraints: None	•	Consistency across models: Yes⸻nvironmental predictorsClimatic variables	•	Source: TerraClimate	•	Variables:	•	Monthly maximum temperature	•	Monthly minimum temperature	•	Monthly vapour pressure deficit	•	Spatial resolution: ~4 kmPreprocessing	•	Monthly time series were transformed using harmonic (Fourier) regression	•	The first three coefficients per variable were retained as predictors	•	Total climatic predictors: 9Collinearity treatment	•	Not applied, as Fourier coefficients are orthogonal by constructionScaling	•	Not applied⸻dditional predictors (model-dependent)	•	Modelled distributions of tick species (82 species)	•	Tick chorotypes	•	Vertebrate chorotypes	•	Distribution of human-biting tick species	•	Livestock density	•	Climatic predictors (as above)⸻ — ModelAlgorithms used	•	Random Forest	•	Multivariate Adaptive Regression Splines (MARS)	•	Generalised Additive Mixed Models (GAMM)	•	Support Vector Machines (SVM)	•	Maximum Entropy (Maxent, implemented via maxnet)⸻mplementation details	•	Framework: tidymodels (for RF, MARS, GAMM, SVM) and maxnet (for Maxent)	•	Hyperparameters:	•	Maxent: feature classes and regularisation parameters (standard settings or as defined in prior calibration workflows, e.g. Wallace/ENMeval)	•	Other models: tuned within the tidymodels framework (details model-specific)	•	Data partitioning:	•	Training/testing split: 70% / 30%	•	Cross-validation:	•	Random resampling (if applied within tuning procedures)⸻ — AssessmentEvaluation metrics	•	Area Under the Curve (AUC)	•	True Skill Statistic (TSS)	•	Cohen’s Kappa	•	Accuracy	•	Calibration slope	•	Omission rateThreshold selection	•	Maximum (sensitivity + specificity)Validation scheme	•	Random train/test split⸻ — Prediction	•	Model output: Continuous suitability values (0–1)	•	Spatial output format: Raster predictions (SpatRaster, implemented via terra)	•	Projection domain: Current climatic conditions only	•	Extrapolation control: Not applied
