# Supplementary figures and images for: Crimean–congo haemorrhagic fever virus circulates within broad ecological networks of ticks and vertebrates
Source: PLoS Negl Trop Dis. 2026 May 27;20(5):e0013783. doi: 10.1371/journal.pntd.0013783 (PMC13232941; doi:10.1371/journal.pntd.0013783)

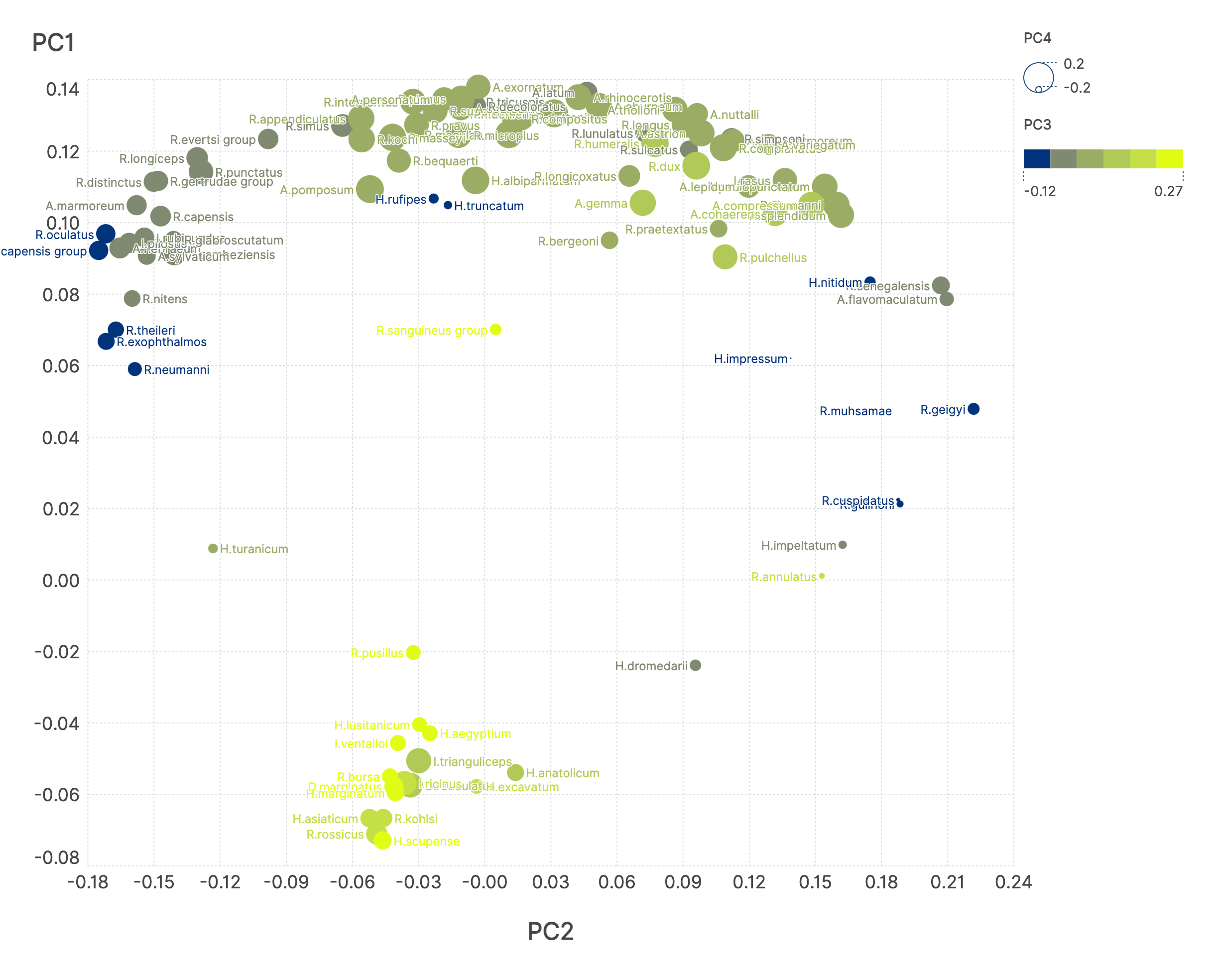

Supplement: S1 Fig — Dots are placed along the coordinates of chorotypes 1 and 2, with colour explaining the ordination in the chorotype 3. The chart intends to be informative but not exhaustive as some points are very near in the reduced space and their labels could not be separated. (PNG) [file pntd.0013783.s006.png]

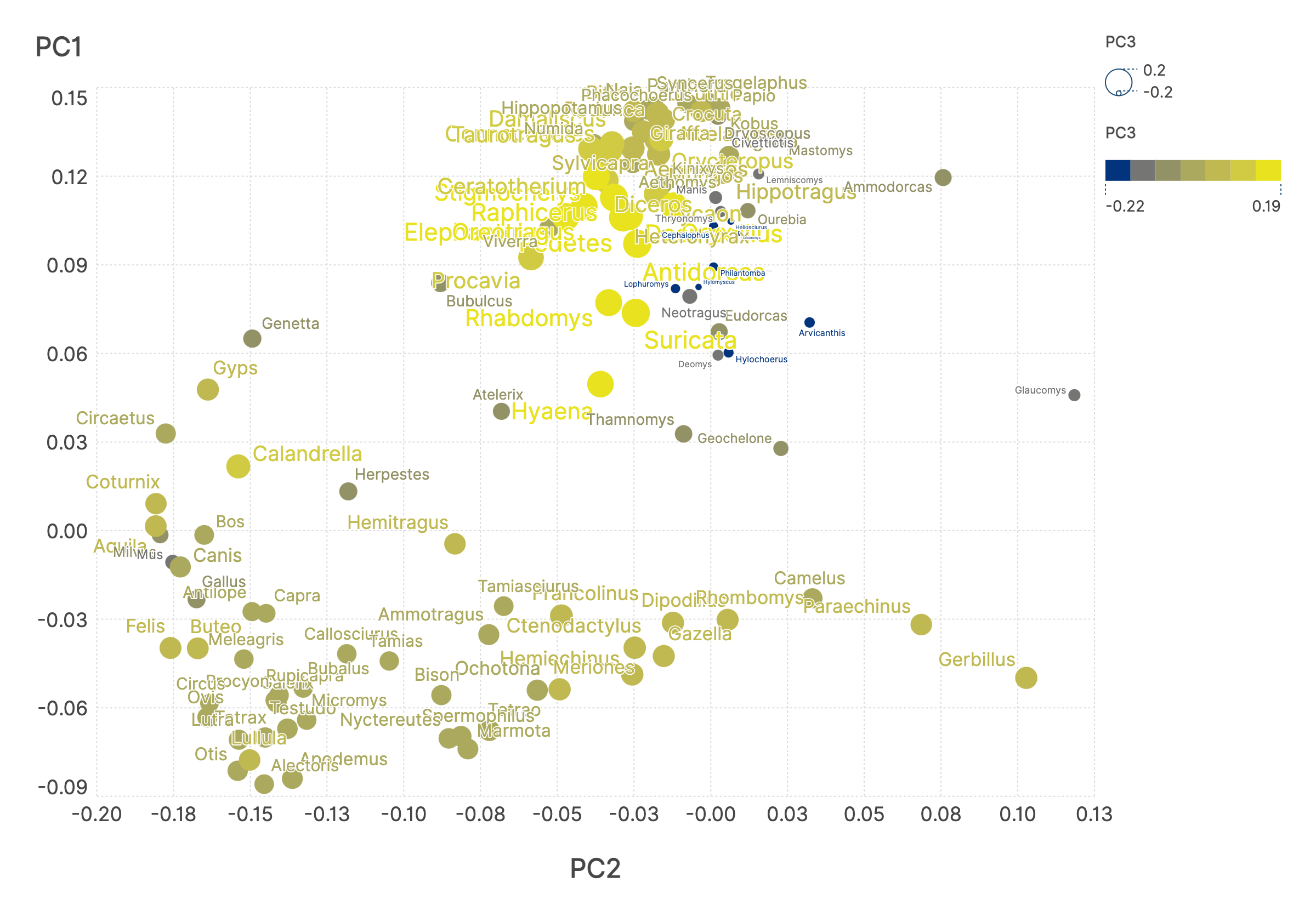

Supplement: S2 Fig — Dots are placed along the coordinates of chorotypes 1 and 2, with colour explaining the ordination in the chorotype 3. The chart intends to be informative but not exhaustive as some points are near in the reduced space and their labels could not be separated. (PNG) [file pntd.0013783.s007.png]
